# The coprecipitation-functionalized 3D-printed GM/PDA/PRF hydrogel for infected bone regeneration via synergistic photothermal antibacterial and osteogenic activity

**DOI:** 10.1016/j.mtbio.2026.103233

**Published:** 2026-05-14

**Authors:** Zihang Yu, Lei Miao, Shuchang Liu, Lijun Zhang, Xiaoqing Wang, Yujia Han, Ziwen Cao, Fan Liu, Zhongti Zhang, Yulou Tian

**Affiliations:** a Department of Orthodontics, School and Hospital of Stomatology, Liaoning Provincial Key Laboratory of Oral Diseases, China Medical University, No. 117 Nanjing North Street, Shenyang, Liaoning, 110002, PR China; b Department of Periodontics, School and Hospital of Stomatology, Liaoning Provincial Key Laboratory of Oral Diseases, China Medical University, No. 117 Nanjing North Street, Shenyang, Liaoning, 110002, PR China; c The VIP Department, School and Hospital of Stomatology, Liaoning Provincial Key Laboratory of Oral Diseases, China Medical University, No. 117 Nanjing North Street, Shenyang, Liaoning, 110002, PR China

**Keywords:** Infected bone repair, 3D printing, Coprecipitation, Polydopamine, Photothermal antibacterial

## Abstract

To address insufficient osteogenesis and persistent infection in infected bone defects, we developed a dual-functionalized hydrogel by integrating polydopamine (PDA) and platelet-rich fibrin (PRF) into a 3D-printed gelatin methacryloyl/methylcellulose (GM) matrix. Two functionalization strategies, surface adsorption and coprecipitation, were employed to fabricate GM/PRF-A and GM/PRF-C scaffolds. Comprehensive characterization revealed both scaffolds exhibited favorable hydrophilicity, controllable degradation and improved mechanical properties. The incorporation of PDA within hydrogels displayed NIR-responsive photothermal antibacterial activity, as well as serving as a bio-adhesive to stabilize PRF and enable on-demand growth-factor release. Both functionalization strategies promoted the proliferation and osteogenic differentiation of bone marrow mesenchymal stem cells. Compared with the adsorption-based scaffold, the coprecipitation-functionalized hydrogel showed more sustained growth-factor release, better antibacterial efficacy, and superior osteogenic performance *in vitro* and in an infected calvarial defect model. These findings indicated that coprecipitation-functionalized GM/PDA/PRF hydrogel was a promising strategy for infected bone regeneration.

## Highlights

• The 3D-printed GM/PDA/PRF scaffold prepared by coprecipitation outperforms surface adsorption in terms of sustained release and osteogenic function.
• NIR-triggered mode switching enables sequential antibacterial and pro-osteogenic functions.
• PDA integrates photothermal antibacterial effects with on-demand release of osteogenic factors.
• Key pathways including BMP and PI3K-Akt are activated to synergistically promote bone regeneration.

## Graphical abstract

**Figure:**
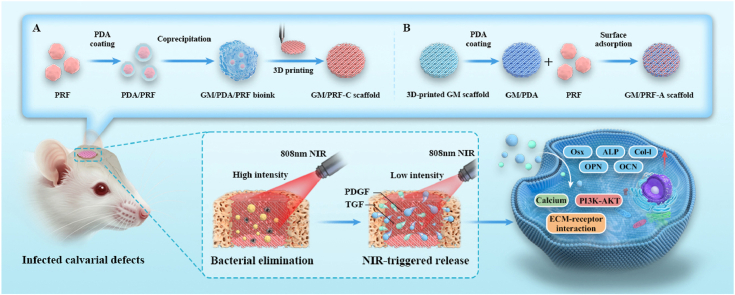

## 1 Introduction

Bone defects resulting from trauma, tumor resection, or infection often lead to severe functional impairment, representing a significant clinical challenge [1]. Infected bone defects are particularly difficult to manage due to their persistent infection and complex pathophysiology. Epidemiological data indicates that postoperative infection rate after internal fixation of open fractures exceeds 30%, with treatment costs in the United States averaging $150,000 per patient [2]. Current clinical options such as autografts and allografts are limited by donor scarcity, donor site morbidity, immune rejection and the need for extra surgeries [3]. Consequently, the development of synthetic bone substitutes has become increasingly critical, particularly for the regeneration of infected bone.

Three-dimensional (3D) printing has emerged as a promising technology in bone tissue engineering, enabling the fabrication of scaffolds with patient-specific geometries and complex architectures [[4], [5], [6]]. Gelatin methacryloyl (GelMA), a photopolymerizable hydrogel, has been widely used in 3D printing owing to its high biocompatibility and bioactivity [[7], [8], [9]]. However, its practical utility is still limited by low viscosity and inadequate shear-thinning behavior at physiological temperature [10]. To address these issues, methylcellulose (MC) can be incorporated as a rheological modifier, yielding a GelMA/MC (GM) hydrogel with enhanced printability, shape fidelity and mechanical stability [11,12]. Nevertheless, GM hydrogel lacks inherent osteoinductive and antibacterial properties, restricting their application in infected bone repair.

The incorporation of bioactive components is essential for effective regeneration [13,14]. Platelet-rich fibrin (PRF), an autologous fibrin matrix enriched with growth factors such as platelet-derived growth factor (PDGF), transforming growth factor beta (TGF-β) and vascular endothelial growth factor, has attracted considerable attention for its osteogenic effect [15,16]. However, its clinical translation remains limited by poor stability and unpredictable release kinetics [[17], [18], [19]]. Polydopamine (PDA), a bioinspired polymer, exhibits strong adhesive properties through its catechol groups, enabling stable immobilization of PRF and sustained growth-factor retention [16,[20], [21], [22]]. Beyond its adhesive function, PDA also shows excellent photothermal conversion efficiency under near-infrared (NIR) irradiation, enabling antibacterial treatment at higher power and pro-osteogenic stimulation under mild hyperthermia (40-42 °C) [[23], [24], [25], [26], [27]]. Despite these advantages, more attention should be paid to how different PDA-based functionalization strategies affect the retention of bioactive components and the overall regenerative performance of the scaffold.

The strategy for immobilizing bioactive agents critically determines the efficacy of engineered scaffolds [15,18]. Among the commonly used approaches, surface adsorption and coprecipitation each have distinct advantages and limitations, and their effectiveness largely depends on specific material design. Previous studies have shown that these two methods can lead to different loading behaviors, release profiles and biological outcomes, indicating that the mode of incorporation is an important determinant of scaffold function [28,29]. Therefore, the primary objective of this study was to directly compare these two strategies in order to clarify their influence on scaffold performance and provide a rational basis for the design of multifunctional hydrogels for infected bone regeneration.

Herein, we developed two dual-functionalized 3D-printed scaffolds: GM/PRF-A (surface adsorption) and GM/PRF-C (coprecipitation). The scaffolds were characterized for physicochemical properties, growth-factor release and photothermal antibacterial performance against *Staphylococcus aureus* (*S. aureus*) and *Escherichia coli* (*E. coli*). *In vitro* studies using bone marrow mesenchymal stem cells (BMSCs) evaluated biocompatibility and osteogenic differentiation, and the underlying osteogenic mechanism by transcriptome analysis. Additionally, an infected rat calvarial defect model was established to assess *in vivo* antibacterial efficacy, biosafety and bone regeneration.

## 2 Experimental section

### 2.1 Materials

Lithium phenyl-2,4,6-trimethylbenzoylphosphinate (LAP) and GelMA (EFL-GM-60, degree of substitution: 60 ± 5%; molecular weight: 100-200 kDa; turbidity: ≤20 NTU) were purchased from Yongqinquan Intelligent Equipment Co., Ltd. (Suzhou, China). Rat BMSCs (sc20230517) were supplied by Fenghui Biotechnology Co., Ltd. (Hunan, China). *S. aureus* (ATCC 25923), *E. coli* (ATCC 25922) and *methicillin-resistant S. aureus* (MRSA, ATCC 33591) were used for antibacterial evaluation. Fetal bovine serum was acquired from TianHang Biotechnology Co., Ltd. (Zhejiang, China). Alpha minimum essential medium, nutrient broth, nutrient agar, MC (M8070, viscosity grade: 4000 cP), dopamine hydrochloride (D9520) and crystal violet staining solution (G1062) were obtained from Beijing Solarbio Science & Technology Co., Ltd. (Beijing, China). The following kits were purchased from Beyotime Biotechnology Co., Ltd. (Shanghai, China): alkaline phosphatase, BCA protein assay, BCIP/NBT alkaline phosphatase color development, Calcein-AM/PI live/dead viability, CCK-8, Actin-Tracker Red-555, DAPI and Alizarin Red S. Enzyme-linked immunosorbent assay (ELISA) kits for PDGF-BB (E-EL-R0537) and TGF-β1 (E-EL-0162) were sourced from Elabscience Biotechnology Co., Ltd. (Wuhan, China). All reagents were used as received unless otherwise stated. RNA extraction was performed using the RNA-quick purification kit (RN001, Yishan Biotechnology, Shanghai).

### 2.2 Preparation of the composite hydrogels

PRF was obtained by centrifuging blood collected from the abdominal aortas of rats at 3000 rpm for 10 min. The translucent intermediate layer, identified as PRF, was carefully separated and processed into freeze-dried powder for subsequent experiments.

A PDA solution (0.1 wt%) was prepared by dissolving dopamine hydrochloride in Tris-HCl buffer (10 mM, pH 8.5). PRF powder was then added to the PDA solution at a concentration of 1 wt% and stirred continuously at 37 °C for 24 h. Unbound components were removed via repeated centrifugation and washing, followed by homogenization through ultrasonic dispersion. The resulting PDA/PRF composite was lyophilized into powder.

The GM hydrogel precursor was prepared by dissolving lyophilized GelMA in PBS containing 0.25 wt% LAP, followed by the incorporation of 8 wt% MC under stirring for 30 min. Scaffolds were fabricated using a desktop 3D bioprinter (SunP BioMarker 2i, China) with the following parameters: a 400 μm nozzle, 120 kPa pressure, 4 °C cartridge temperature, 2 mm/s printing speed, 1 mm line spacing, and 0.3 mm layer height. The printed structure was UV-crosslinked (25 mW/cm^2^) for 60 s to form stable GM hydrogel.

For surface-functionalized scaffolds (GM/PRF-A), GM scaffolds were immersed in a dopamine solution (1 mg/mL) at 4 °C overnight to form a PDA coating, followed by adsorption of 1 wt% PRF under the same conditions. For coprecipitated scaffolds (GM/PRF-C), 1 wt% PDA/PRF powder was blended into the GM precursor solution via magnetic stirring at 37 °C for 3 h before printing and crosslinking.

All samples were lyophilized using a freeze dryer (SCIENTZ-12N/B, China) and sterilized using cobalt-60 irradiation (15 kGy, 20 h) prior to use.

### 2.3 Physicochemical characterization

The surface and cross-sectional morphologies of the lyophilized scaffolds were examined by scanning electron microscopy (SEM, Zeiss GeminiSEM 300, Germany). Pore size and porosity were quantified using ImageJ software. To further verify element composition and distribution, EDS spectra and elemental mapping were collected for GM, GM/PRF-C and GM/PRF-A.

The chemical structures of the scaffolds were analyzed by Fourier transform infrared spectroscopy (FTIR, Thermo Fisher Scientific Nicolet iS20, USA; 400-4000 cm^−1^), X-ray diffraction (XRD, Rigaku SmartLab SE, Japan), and X-ray photoelectron spectroscopy (XPS, Thermo Scientific ESCALAB 250Xi, USA).

Rheological properties were characterized with a rheometer (Anton Paar MCR 302, Austria) by frequency sweep (0.1-10 Hz) to determine storage modulus (G′) and loss modulus (G″). Surface hydrophilicity was evaluated by water contact angle measurement. The swelling ratio was determined by immersing lyophilized scaffolds (W_d_) in PBS at 37 °C. At 1, 2, 4, 6, 8, 10 and 12 h, samples were removed, blotted and weighed (W_s_). The water absorption capacity was calculated as:

(1) $$W_{a}=\frac{W_{s}‐W_{d}}{W_{d}}\times 100\%$$

For *in vitro* degradation, pre-swollen scaffolds at equilibrium (W_0_) were incubated in PBS or in *S. aureus* suspension (1 × 10^6^ CFU/mL) at 37 °C. The bacterial suspension was refreshed every 2 days. Samples were weighed (W_t_) at 1, 2, 3, 5, 7, 14, 21 and 28 days, and the degradation ratio was calculated as:

(2) $$\text{Degradation}\;\text{ratio}\;\left(\%\right)=\frac{W_{0}‐W_{t}}{W_{0}}\times 100\%$$

Compressive properties were measured using a mechanical testing machine (Hengyi, China) at 1 mm/min until 30% strain, and stress-strain curves were recorded. Furthermore, to assess mechanical stability under infection-relevant conditions, scaffolds incubated in *S. aureus* suspension were collected at 0, 1, 3, 7 and 14 days, gently rinsed with PBS, and tested using the same compression protocol.

To evaluate surface stability during degradation, pH was monitored in PBS (0.01 M, pH 7.4) at 37 °C. The pH of the degradation medium was measured at 0 h, 1 h, 6 h, 1, 3, 5, 7, 14, 21 and 28 days. PBS was replenished after each measurement to maintain a constant degradation environment. For zeta potential analysis, hydrogel samples were collected at 0, 1, 7, 14 and 28 days, gently rinsed, fragmented, dispersed in PBS, and measured using a Zetasizer Nano ZS90 (Malvern Panalytical, UK).

Photothermal performance was evaluated using an 808 nm NIR laser. Temperature changes under irradiation were recorded with an infrared thermal camera (Hikmicro, China) for 5 min. The cooling curves were collected after the laser was turned off.

PDGF-BB and TGF-β1 were used as representative PRF-derived factors [30,31]. Their release from PRF powder, GM/PRF-C and GM/PRF-A, with or without NIR irradiation, was quantified by ELISA. Samples were incubated in PBS (pH 7.4) at 37 °C and irradiated for 5 min on days 3, 7 and 11. Supernatants were collected at 1, 2, 3, 5, 7, 9, 11 and 14 days, and replaced with fresh PBS after each sampling.

### 2.4 *In vitro* antibacterial assessment

*S. aureus*, *E. coli* and MRSA were used to evaluate the antibacterial properties of the composite hydrogels. Six groups were established: Control, GM, GM/PRF-A, GM/PRF-C, GM/PRF-A + NIR and GM/PRF-C + NIR. Briefly, 200 μL of bacterial suspension (1 × 10^6^ CFU/mL) was added onto each sterilized sample and incubated at 37 °C for 4 h. For the NIR groups, an 808 nm laser at 2 W/cm^2^ was applied for 5 min during incubation.

After incubation, samples were ultrasonically treated for 2 min to detach bacteria. The suspension was serially diluted, plated on agar, and incubated at 37 °C for 24 h before colony counting. Antibacterial rate was calculated as follows:

(3) $$\text{Antibacterial}\;\text{rate}\;\left(\%\right)=\frac{\text{CFU}\;\text{of}\;\text{control}\;\text{grou}p‐\text{CFU}\;\text{of}\;\text{experimental}\;\text{group}}{\text{CFU}\;\text{of}\;\text{control}\;\text{group}}\times 100\%$$

Bacterial growth in the supernatant was further evaluated by measuring OD_600_. Bacterial morphology on the scaffold surface was observed by SEM after fixation with 2.5% glutaraldehyde, graded ethanol dehydration. Gold sputter coating was prepared before SEM observation.

Biofilm formation of *S. aureus*, *E. coli* and MRSA was assessed by crystal violet staining. After treatment, samples were gently washed with PBS, stained with 0.1% crystal violet for 15 min, rinsed thoroughly, air-dried and photographed. For quantification, the bound dye was dissolved in 33% acetic acid and measured at OD_590_.

### 2.5 *In vitro* cell analysis

#### 2.5.1 Cell culture

BMSCs were cultured in growth medium consisting of BMSCs basal medium supplemented with 10% FBS and 1% penicillin-streptomycin at 37 °C. The medium was refreshed every three days until cells reached approximately 90% confluence. Cells at passages 3 were used for all subsequent experiments. Prior to cell seeding, all samples were immersed in PBS solution for 8 h, and washed with sterile distilled water.

#### 2.5.2 Biocompatibility evaluation

Five groups (GM, GM/PRF-C, GM/PRF-A, GM/PRF-C + NIR and GM/PRF-A + NIR) were used for hemocompatibility and cytocompatibility testing. For hemolysis, sample extracts were incubated with rat red blood cells. Normal saline and deionized water served as the negative and positive controls, respectively. After centrifugation, supernatant absorbance was measured at 545 nm and hemolysis was calculated using the standard formula.

(4) $$\text{Hemolysis}\;\text{ratio}\;\left(\%\right)=\frac{\text{ODsample}\;‐\;\text{ODnegative}}{\text{ODpositive}\;‐\;\text{ODnegative}}\times 100\%$$

For cytocompatibility, BMSCs were seeded onto the five groups. The NIR groups were irradiated (808 nm, 2 W/cm^2^, 5 min) on days 1, 3, 5 and 7. Cell proliferation was assessed by CCK-8 on days 1, 3, 5 and 7. Cell viability and morphology were evaluated on day 3 by Live/Dead staining and Phalloidin/DAPI staining, respectively. Images were captured by confocal laser scanning microscopy.

### 2.6 *In vitro* osteogenic differentiation assay

BMSCs were cultured in a transwell system with hydrogels placed in the upper chamber. The GM/PRF-C + NIR and GM/PRF-A + NIR groups received NIR irradiation (808 nm, 1 W/cm^2^, 5 min) on days 3, 7 and 11. ALP staining and ALP activity assay were performed on day 7. Mineralization was assessed by ARS staining on day 14, followed by quantification with 10% cetylpyridinium chloride.

Quantitative reverse transcription PCR (qRT-PCR) was performed to investigate osteogenesis-related gene expression. BMSCs were seeded onto the hydrogel with or without NIR laser irradiation (808 nm, 1 W/cm^2^, 5min). On day 7, total RNA was extracted from BMSCs, reverse-transcribed into cDNA, and analyzed by qRT-PCR using SYBR Green Master Mix on a QuantStudio3 system (Applied Biosystems, USA). The expression levels of the osteogenic genes (osterix (Osx), collagen I (Col I), osteopontin (OPN) and osteocalcin (OCN)) were normalized to that of GAPDH. Primer sequences were listed in Table S1.

#### 2.6.1 Transcriptome analysis

After 7 days of culture on the control and GM/PRF-C + NIR hydrogels, total RNA was extracted from BMSCs using Trizol reagent. RNA sequencing was performed by Science Compass (Hangzhou Yanqu Information Technology Co., Ltd., China). Differential gene expression analysis was conducted based on uncorrected *P* values and fold change. Genes with an adjusted *P* value < 0.05 and an absolute fold change >2 after multiple testing correction were considered as significantly expressed.

### 2.7 *In vivo* bone formation ability

#### 2.7.1 Surgical procedures

All animal procedures were approved by the Animal Ethics Committee of China Medical University (No. CMU20251091). An infected calvarial defect model was established in 8-week-old male Sprague-Dawley rats (180-200 g). A 5 mm critical-sized defect was created and inoculated with 1.5 μL of *S. aureus* suspension (1 ×10^8^ CFU/mL). Rats were randomly assigned to four groups: Control (empty defect), GM, GM/PRF-C + NIR and GM/PRF-A + NIR. NIR irradiation (808 nm, 2 W/cm^2^, 5 min) was applied on postoperative days 1, 2 and 3 (Fig. 7A). Two weeks postoperatively, NIR irradiation (808 nm, 1 W/cm^2^, 5 min) was continued once a week.

#### 2.7.2 Biosafety *in vivo*

At 8 weeks after implantation, blood samples were collected for serum biochemical analysis, including alanine aminotransferase (ALT), aspartate aminotransferase (AST), blood urea nitrogen (BUN) and creatinine (CREA). Major organs (heart, liver, spleen, lung and kidney) were harvested, fixed, embedded, sectioned, and stained with hematoxylin and eosin (H&E).

For local biosafety assessment, soft tissues surrounding the defect were collected at 1, 4 and 8 weeks after surgery. H&E staining was performed to evaluate possible thermal injury after repeated irradiation.

#### 2.7.3 Micro-CT analysis

Block sections including the implanted hydrogels and surrounding tissues were collected, fixed in a 10% neutral formalin solution for 14 days, and prepared for imaging using a micro-CT scanning system (SkyScan 1276, Bruker, Germany). Indices, including bone volume fraction (BV/TV), trabecular thickness (Tb.Th) and bone surface fraction (BS/BV), were quantified to evaluate the newly formed bone within the region of interest.

#### 2.7.4 Histological staining

At 1-week post-implantation, granulation tissues harvested from the defect sites were used for antibacterial assessment by spread plate assay. The specimens were further processed for H&E and Giemsa staining.

At 4 and 8 weeks, harvested bone tissues were decalcified, paraffin-embedded, and sectioned for H&E, Masson's trichrome and OCN immunohistochemical staining. All stained sections were examined under a light microscope.

### 2.8 Statistical methods

All data were expressed as mean ± standard deviation from at least three independent replicates. Statistical significance was analyzed using one-way analysis of variance followed by Tukey's multiple comparison test in GraphPad Prism (version 8.0.2, USA). Statistical significance was defined as *p < 0.05, **p < 0.01.

## 3 Results

### 3.1 Physical-chemical properties

**Fig. 1:**
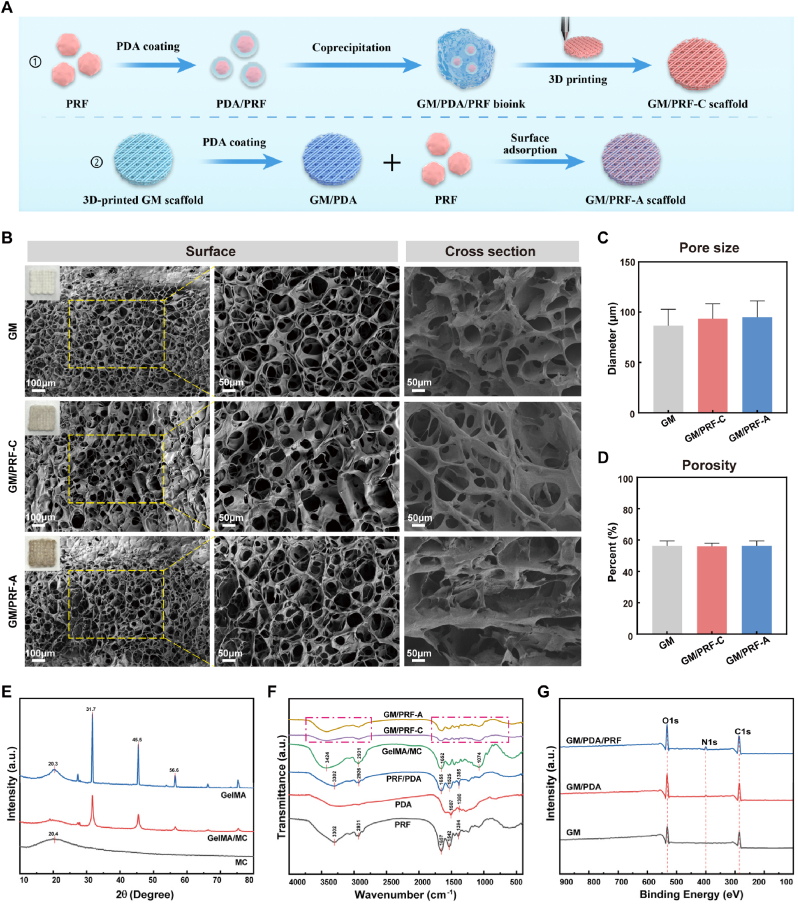
Physicochemical characterization of the composite hydrogels. A) Schematic of the scaffold preparation processes for GM/PRF-C (coprecipitation) and GM/PRF-A (surface adsorption). B) Representative SEM images (surface and cross-sectional morphologies) of the lyophilized GM, GM/PRF-C and GM/PRF-A hydrogels. C, D) Quantitative analyses of pore diameter and porosity from the SEM images. E-G) XRD patterns, FTIR spectra and XPS spectra of GM, GM/PRF-C and GM/PRF-A hydrogels (Scale bars = 100 μm (overview) and 50 μm (high magnification)). (*p < 0.05, **p < 0.01, N = 3).

The optimal PDA and PRF contents (PRF/PDA ratios) were first determined by cell viabilities and ALP activities of BMSCs cultured on the hydrogel matrices (Figs. S1 and S2). It was identified 0.1% PDA and 1% PRF as the optimal ratio. Accordingly, the representative image of printed composite scaffolds (0.1% PDA and 1% PRF) was shown in Fig. S3. Pure GM appeared translucent, whereas GM/PRF-C exhibited a homogeneous brown-black color, and GM/PRF-A showed deeper surface coloration, consistent with PDA enrichment on the outer layer. SEM revealed that all groups maintained a porous honeycomb-like architecture with pore sizes of approximately 60-120 μm (Fig. 1B–D). No significant differences in pore diameter or porosity were observed, indicating that the incorporation of PDA and PRF did not compromise the scaffold microstructure.

XRD, FTIR, XPS and EDS were used to characterize the structure and composition of composite scaffolds (Fig. 1E–G and Fig. S5). XRD analysis (Fig. 1E) showed that MC exhibited a characteristic peak at 20.4°, while GelMA displayed a broad peak at 20.3° with reflections at 31.7°, 45.5° and 56.6°. The GM hydrogel retained the characteristic peaks of both components, indicating preservation of their crystalline features after physical blending. FTIR spectra (Fig. 1F) further confirmed the interactions among PDA, PRF and GM. PRF exhibited typical amide peaks at 3302, 2931, 1657, 1542 and 1394 cm^−1^, whereas PDA exhibited broad O-H/N-H stretching at 3100-3400 cm^−1^ and an aromatic C=C vibration at 1507 cm^−1^. In the PDA/PRF composite, the retention of PRF peaks, the shift of amide Ⅱ from 1542 to 1525 cm^−1^, broadening of amide Ⅰ and reduced N-H stretching suggested interactions between PRF and PDA. The GelMA/MC spectrum had no obvious peak shift, indicating unchanged polymer composition after blending (Fig. S4). Both GM/PRF-A and GM/PRF-C displayed the characteristic signals of PRF/PDA and GelMA/MC, confirming successful incorporation of the functional components. XPS analysis (Fig. 1G) further verified PDA introduction, as an N1s peak was detected in GM/PDA but not in GM, while GM/PDA/PRF showed enhanced N1s and O1s signals. Consistently, EDS mapping (Fig. S5) exhibited stronger surface N and P signals in GM/PRF-A, whereas GM/PRF-C exhibited a surface elemental profile closer to GM, supporting surface-localized loading in GM/PRF-A and more uniform internal distribution in GM/PRF-C.

**Fig. 2:**
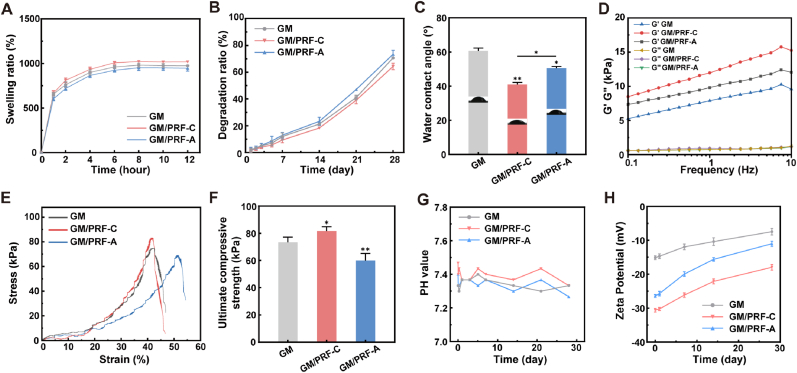
Physicochemical properties of the composite hydrogels. A) Swelling kinetics. B) *In vitro* degradation profiles. C) Water contact angles. D) Rheological properties. E) Compressive stress-strain curves. F) Compressive strength. G) pH values. H) Zeta potential. (*p < 0.05, **p < 0.01, N = 3).

All scaffolds exhibited rapid swelling during the first 4 h, and reached equilibrium by 8 h (Fig. 2A). GM/PRF-C exhibited a slightly higher equilibrium swelling ratio than GM and GM/PRF-A. Contact angle measurements further confirmed the superior hydrophilicity of GM/PRF-C (40.93 ± 1.27°) compared with GM/PRF-A (50.50 ± 1.12°) and GM (60.67 ± 1.63°) (Fig. 2C). Rheological analysis confirmed solid-like behaviors in all groups, with G′ consistently higher than G″ (Fig. 2D). The incorporation of PDA/PRF had minimal impact on G″ but notably enhanced the elastic modulus (G′), indicating it enhanced elastic modulus without compromising viscoelasticity.

Degradation and mechanical stability were further examined under both PBS and bacteria-containing conditions. In PBS, all groups exhibited a relatively slow degradation phase during the first 14 days, followed by accelerated degradation up to day 28 (Fig. 2B). At day 28, GM/PRF-C displayed the lowest degradation ratio (64.33 ± 2.52%), compared with GM/PRF-A (73.33 ± 3.06%) and GM (70.67 ± 0.58%). A similar trend was observed in *S. aureus* suspension, where GM/PRF-C also exhibited the lowest cumulative degradation at day 28 (75.00 ± 2.65%), while GM and GM/PRF-A reached 81.67 ± 1.52% and 86.33 ± 2.52%, respectively (Fig. S6A). In terms of mechanical properties, GM/PRF-C exhibited the highest initial compressive strength (81.67 ± 3.17 kPa), whereas the surface adsorption strategy led to reduced mechanical strength under normal condition (Fig. 2E and F). In the bacteria-containing environment, GM/PRF-C retained the highest residual compressive modulus during the test period, which was 47.0 ± 2.65 kPa on day 14, compared with 36.0 ± 3.61 kPa for GM and 30.33 ± 3.51 kPa for GM/PRF-A (Fig. S6B).

During degradation in PBS, the pH of all groups remained stable within 7.2-7.5 (Fig. 2G). The result of zeta potential revealed GM/PRF-C retained a more negative surface potential on day 28 (−20 ± 3 mV) than GM (−7.44 ± 0.99 mV) and GM/PRF-A (−11.02 ± 0.79 mV), suggesting that coprecipitation better preserved the surface characteristics of the scaffold during degradation (Fig. 2H).

### 3.2 Photothermal capabilities

**Fig. 3:**
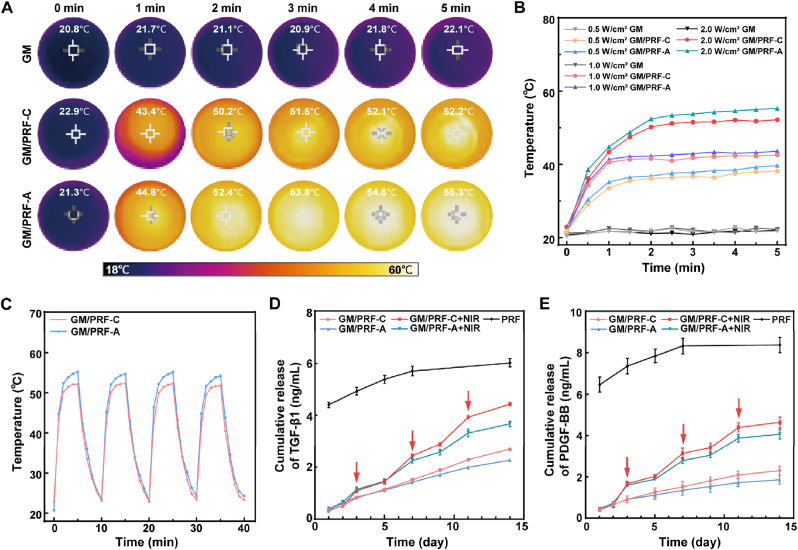
Photothermal performance and growth factors release profiles of the composite hydrogels. A) Representative infrared thermal images of GM, GM/PRF-C and GM/PRF-A hydrogels under NIR irradiation (808 nm, 2.0 W/cm^2^). B) Photothermal heating curves recorded during NIR irradiation at 0.5, 1.0 and 2.0 W/cm^2^ for 5 min. C) Photothermal stability of GM/PRF-C and GM/PRF-A hydrogels over four on/off NIR cycles (808 nm, 2.0 W/cm^2^). D, E) Cumulative release profiles of TGF-β1 and PDGF-BB from GM/PRF-C and GM/PRF-A hydrogels for 14 days. Red arrows indicate time points of NIR irradiation (days 3, 7 and 11). (*p < 0.05, **p < 0.01, N = 3). (For interpretation of the references to color in this figure legend, the reader is referred to the Web version of this article.)

As illustrated in Fig. 3A–C, the GM hydrogel without PDA showed negligible temperature change, whereas both GM/PRF-C and GM/PRF-A displayed rapid photothermal responses under NIR irradiation. GM/PRF-C increased more gradually and stabilized at about 52 °C after 5 min, while GM/PRF-A occasionally exceeded 55 °C. Both functionalized hydrogels maintained stable heating over multiple on-off cycles. To further select optimal irradiation parameters for biological evaluation, additional multi-power NIR tests were performed at 0.5, 1.0 and 2.0 W/cm^2^. As shown in Fig. 3B and S7, 1.0 W/cm^2^ generated mild hyperthermia which was suitable for osteogenesis-related stimulation, while 2.0 W/cm^2^ reached the temperature range required for antibacterial treatment [32,33]. Accordingly, 1.0 W/cm^2^ was used for osteogenic experiments and 2.0 W/cm^2^ for antibacterial experiments.

### 3.3 Growth factors release pattern

PDGF-BB and TGF-β1 were quantified as representative PRF-derived factors (Fig. 3D and E). All groups showed an initial burst release on day 1, consistent with typical diffusion-driven release from hydrogel matrices. GM/PRF-C + NIR and GM/PRF-A + NIR groups enhanced the release of growth factors under NIR irradiation at each time point (days 3, 7 and 11). This on-demand release behavior was attributed to the photothermally temperature rise, which weakened hydrogen bonding and hydrophobic interactions, thereby promoting growth factors release. In contrast, hydrogels without NIR exposure maintained low and basal release rates throughout the observation period. Compared with GM/PRF-A, GM/PRF-C achieved more prolonged and stable NIR-triggered release. In detail, surface adsorption of GM/PRF-A had weakened interfacial integration due to the rapid depletion of superficially bounding and localized thermal effects, exhibiting a more pronounced decline in release during the later stages. The GM/PRF-C + NIR group achieved the highest cumulative release, indicating the superiority of the coprecipitation approach for the robust and on-demand growth factors delivery system.

### 3.4 Antibacterial performance *in vitro*

**Fig. 4:**
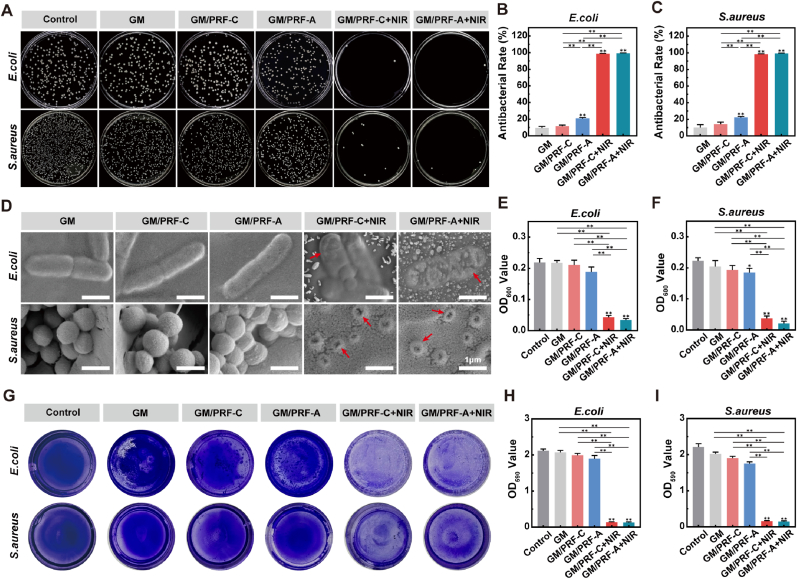
*In vitro* antibacterial properties of the composite hydrogels against *E. coli* and *S. aureus.* A) Representative photographs of bacterial colonies in the presence of composite hydrogels with or without NIR irradiation (2 W/cm^2^, 5 min). B, C) Quantitative antibacterial rates of composite hydrogels with or without NIR irradiation against *E. coli* and *S. aureus*. D) Representative SEM images of bacterial morphology in the presence of composite hydrogels with or without NIR irradiation (scale bar = 1 μm). E, F) Bacterial growth kinetics of *E. coli* and *S. aureus*. G) Crystal violet staining of biofilm. H, I) Quantitative analysis of crystal violet-stained biofilms of *E. coli* and *S. aureus*. (*p < 0.05, **p < 0.01, N = 3). (For interpretation of the references to color in this figure legend, the reader is referred to the Web version of this article.)

The antibacterial performance of the composite hydrogels was first evaluated against *S. aureus* and *E. coli*. As shown in Fig. 4A, both the GM and control groups exhibited high bacterial loads. Without NIR irradiation, GM/PRF-A showed moderate antibacterial activity, probably due to the intrinsic effect of the PDA coating. No differences were found between GM/PRF-C and control group. Under NIR irradiation, both GM/PRF-C and GM/PRF-A exhibited strong antibacterial activity, reaching 98.67 ± 0.58% and 99.33 ± 0.58% against *E. coli*, and 98.33 ± 0.58% and 99.30 ± 0.58% against *S. aureus*, respectively (Fig. 4B and C). Consistently, the OD_600_ values were significantly reduced in the GM/PRF-C + NIR and GM/PRF-A + NIR groups (Fig. 4E and F). SEM images showed severe bacterial damage after NIR treatment, demonstrating their strong photothermal antibacterial effects (Fig. 4D).

Crystal violet staining further indicated that NIR treatment effectively inhibited biofilm formation of both *S. aureus* and *E. coli* (Fig. 4G–I). Dense biofilm accumulation was observed in the control and non-irradiated groups, while GM/PRF-C + NIR and GM/PRF-A + NIR markedly weakened the staining with significantly lower OD_590_ values.

To further enhance clinical relevance, additional tests were performed against MRSA. As shown in Fig. S8, the anti-bacterial efficiencies of GM/PRF-C + NIR and GM/PRF-A + NIR were 99.2 ± 0.5% and 98.7 ± 0.6%, respectively. OD_600_ values were also significantly reduced after NIR treatment. Crystal violet staining revealed greatly reduced biofilm formation compared with the non-irradiated groups. These results indicated that the photothermal antibacterial effect of the composite hydrogels was effective against both standard and drug-resistant strains.

### 3.5 Biocompatibility *in vitro*

**Fig. 5:**
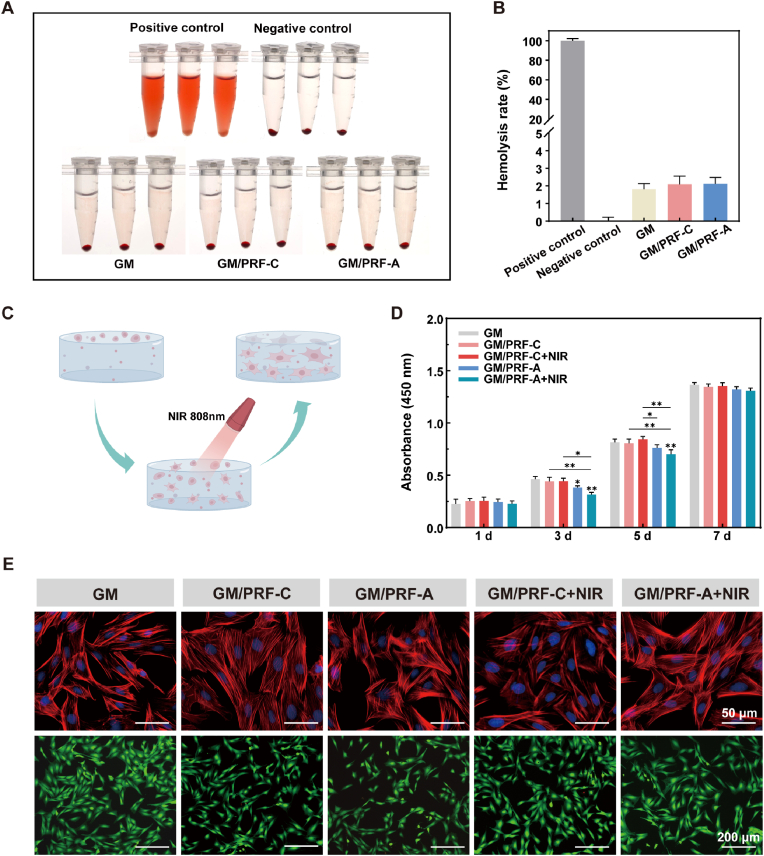
*In vitro* biocompatibility of the composite hydrogels. A) Photographs of hemolysis. B) Hemolysis rates of GM, GM/PRF-C and GM/PRF-A hydrogels. C) Schematic of BMSCs cultured on hydrogels under periodic NIR irradiation. D) Viability of BMSCs seeded onto the scaffolds, assessed by CCK-8 assay on days 1, 3, 5 and 7. E) Cytoskeletal staining (scale bar = 50 μm) and live/dead staining (scale bar = 200 μm) of BMSCs after 3 days of culture on the hydrogels. (*p < 0.05, **p < 0.01, N = 3).

An *in vitro* hemolysis assay was first performed to evaluate blood compatibility. As shown in Fig. 5A and B, the hemolysis rates of GM, GM/PRF-C and GM/PRF-A were 1.82 ± 0.31%, 2.10 ± 0.46% and 2.11 ± 0.37%, respectively, all of which were below 5%, indicating their favorable hemocompatibility.

For cytocompatibility, cell proliferation increased over time in all groups (Fig. 5C and D). Compared with GM, the GM/PRF-A and NIR-treated groups exhibited relatively lower proliferation on days 3 and 5. No significant difference was found among all groups on day 7. Live/Dead staining indicated that most cells remained viable in all groups (Fig. 5E). Additionally, phalloidin staining demonstrated BMSCs cultured on the functionalized scaffolds exhibited a well-spread morphology with extended pseudopodia, displaying typical stem cell morphology (Fig. 5E). These results suggested that PDA and PRF incorporation did not compromise the cytocompatibility of the GM scaffold.

### 3.6 Osteogenesis *in vitro*

**Fig. 6:**
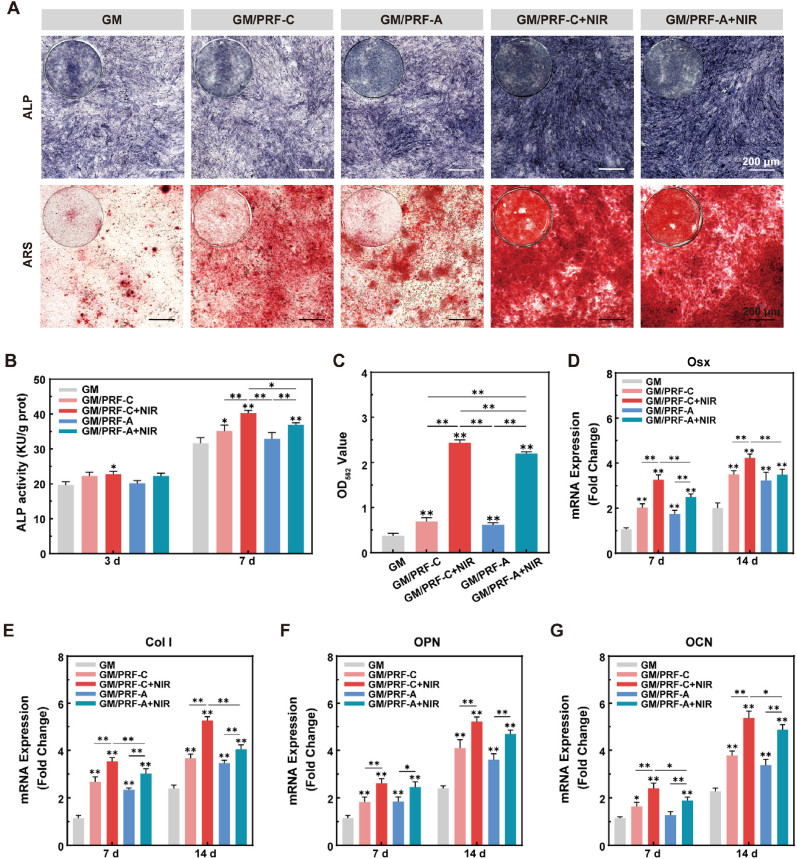
*In vitro* osteogenic properties of the composite hydrogels. A) Representative images of Alkaline phosphatase (ALP) staining (day 7) and Alizarin Red S (ARS) staining (day 14) of BMSCs (scale bar = 200 μm). B) ALP activity of BMSCs after 3 and 7 days of culture. C) Extracellular matrix mineralization quantified after 14 days. (D-G) mRNA expression levels of osteogenic markers (osterix (Osx), collagen I (Col I), osteopontin (OPN), and osteocalcin (OCN)) on day 7 and 14. (*p < 0.05, **p < 0.01, N = 3). (For interpretation of the references to color in this figure legend, the reader is referred to the Web version of this article.)

The osteogenic effect was evaluated by ALP activity and ARS staining. On day 3, GM/PRF-C + NIR significantly enhanced ALP activity compared with GM (Fig. 6B). By day 7, both NIR-treated groups showed higher ALP activity, which was further confirmed by ALP staining (Fig. 6A). Similarly, ARS staining and its semi-quantitative analysis revealed more mineralized nodules in the NIR-treated groups after 14 days. Specifically, GM/PRF-C + NIR group demonstrated the highest degree of mineralization (Fig. 6A and C).

**Fig. 7:**
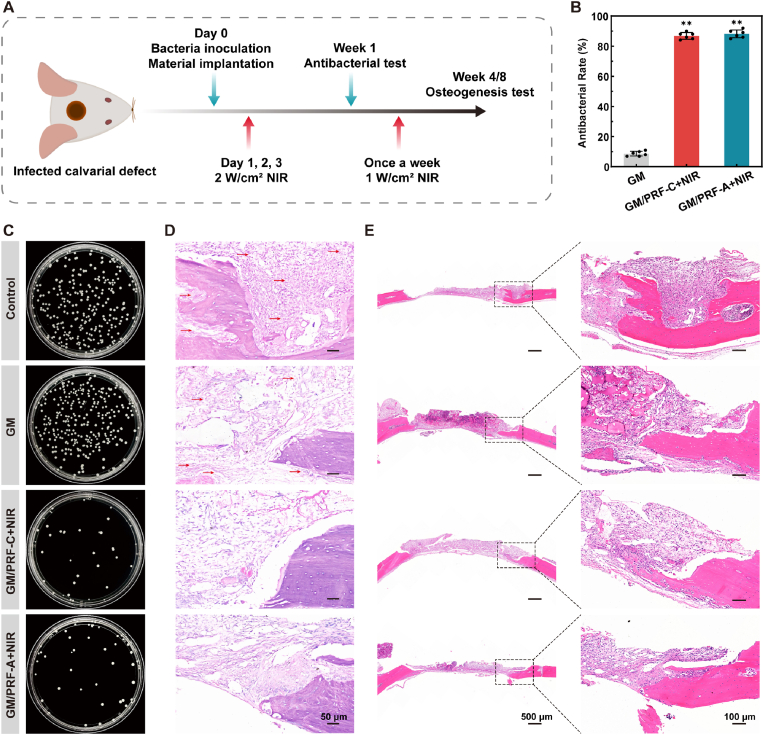
*In vivo* antibacterial effect of the composite hydrogels. A) Experimental design timeline for therapeutic monitoring. B, C) Antibacterial rates against *S. aureus* and its bacterial colonies counts after NIR irradiation *in vivo*. D) Representative images of Giemsa (scale bar = 50 μm) and E) H&E (scale bars = 500 μm for overview, 100 μm for high magnification) staining at one-week post-surgery. Red arrows indicated bacteria in the tissue. (*p < 0.05, **p < 0.01, N = 6). (For interpretation of the references to color in this figure legend, the reader is referred to the Web version of this article.)

qRT-PCR analysis of Osx, Col I, OPN and OCN was shown in Fig. 6D–G. Generally, GM/PDA/PRF with or without NIR irradiation exhibited higher osteogenesis-related gene expressions compared with GM group. GM/PRF-C under NIR irradiation upregulated these osteogenic markers the most, which was significantly higher than the rest of the groups. These results indicated that mild photothermal stimulation synergized with PDA/PRF-derived biochemical cues to promote osteogenic differentiation of BMSCs.

### 3.7 Antibacterial activity *in vivo*

To evaluate antibacterial efficacy *in vivo*, the GM, GM/PRF-C and GM/PRF-A hydrogels were implanted into an infected calvarial defect model (Fig. S9). During antibacterial treatment (808 nm, 2 W/cm^2^), the temperature of the implanted GM/PRF-C and GM/PRF-A increased from about 36 °C to 50 °C within 2 min and stabilized around 55 °C (Fig. S10), indicating effective photothermal heating *in vivo*.

After NIR irradiation, the antibacterial efficiencies of GM/PRF-C and GM/PRF-A reached 86.8 ± 2.2% and 88.17 ± 2.6%, respectively, which were significantly higher than that of GM (8.7 ± 1.6%) (Fig. 7B and C). Giemsa staining showed a significant bacterial reduction in both NIR-treated groups (Fig. 7D). H&E staining revealed substantially reduced inflammatory infiltration compared with the control and GM groups (Fig. 7E). These findings indicated that photothermal treatment effectively controlled infection and created a more favorable microenvironment for defect repair.

### 3.8 Biosafety *in vivo*

Serum biochemical analysis showed that ALT, AST, BUN and CREA remained within the normal range, which had no significant difference from the control group (Fig. S13). Additionally, H&E staining of the heart, liver, spleen, lung and kidney revealed no obvious pathological abnormalities (Fig. S14). Regrading to degradation behavior, the scaffold showed progressive degradation during implantation (Fig. S11). Histological examination of the soft tissues surrounding the defect further identified no evident thermal injury after repeated NIR irradiation, whereas the non-irradiated infected groups exhibited more severe local inflammation (Fig. S12). These findings supported the good systemic and local biosafety of the composite hydrogels *in vivo*.

### 3.9 Osteogenic activity *in vivo*

**Fig. 8:**
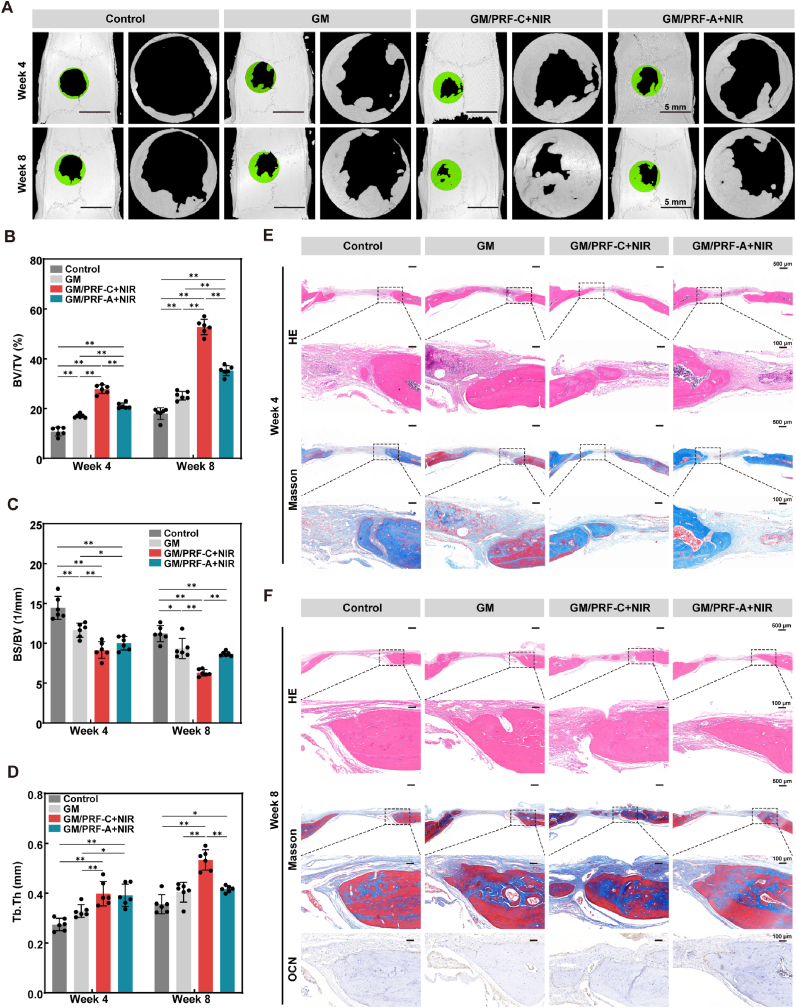
*In vivo* osteogenic effect of the composite hydrogels. A) Representative images of reconstructed micro-CT. B-D) Quantitative analysis of BV/TV, BS/BV and Tb.Th of the micro-CT data. E) Representative images of H&E and Masson staining at week 4 post-surgery. F) Representative images of H&E, Masson's trichrome and immunohistochemical (OCN) staining at week 8 post-surgery. (Scale bars = 500 μm (overview) and 100 μm (high magnification)). (*p < 0.05, **p < 0.01, N = 6).

To evaluate bone regeneration *in vivo*, the defect sites in the GM/PRF-C + NIR and GM/PRF-A + NIR groups were irradiated weekly with an 808 nm laser at 1 W/cm^2^. As shown in Fig. S10, the local temperature increased to 40 °C rapidly, and stabilized between 40 and 42 °C. Micro-CT images at 4 and 8 weeks exhibited minimal healing in the control and GM group, while both NIR-treated groups exhibited significantly enhanced bone regeneration (Fig. 8A). GM/PRF-C + NIR achieved the highest BV/TV (52.76 ± 3.06%) and Tb.Th (0.53 ± 0.04 mm), and the lowest BS/BV (6.32 ± 0.42 mm^−1^) at 8 weeks (Fig. 8B–D). Furthermore, histological analysis was performed on the defect sites using H&E and Masson's trichrome staining (Fig. 8E and F). At 4 weeks, the defect areas in the control and GM groups were predominantly filled with fibrous connective tissue, accompanied by abundant inflammatory cell infiltration. However, the GM/PRF-C + NIR and GM/PRF-A + NIR groups exhibited remarkable neovascularization and new bone formation within the defect regions. By 8 weeks, all groups with implanted scaffolds demonstrated substantial new bone formation. In the GM/PRF-A + NIR group, moderate new bone formation and vascularization were observed at the defect periphery. In the GM/PRF-C + NIR group, extensive new bone tissue and vascular infiltration were evident not only at the periphery but also in the central region of the defect, indicating a more robust bone regeneration. Immunohistochemical staining for OCN at 8 weeks confirmed the enhanced osteogenic activity in the GM/PRF-C + NIR group, indicating active bone matrix maturation [34].

### 3.10 Transcriptome analysis of BMSCs

**Fig. 9:**
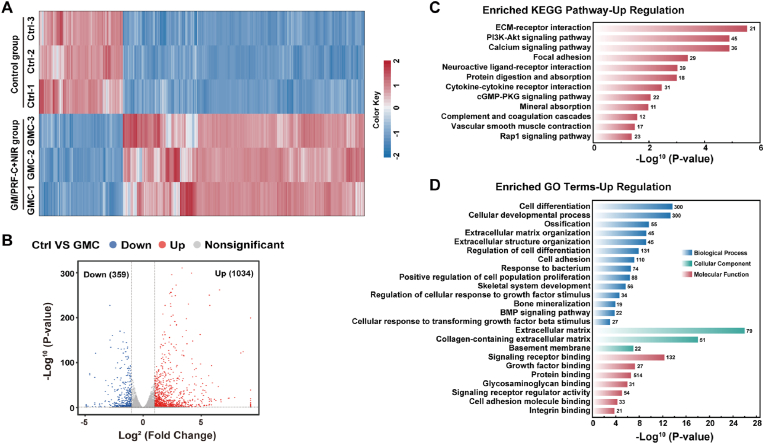
Gene transcriptional analysis of NIR-responsive GM/PRF-C hydrogel in promoting osteogenesis. A) Hierarchical clustering heatmap of RNA-sequencing data. B) Volcano plot visualizing differential gene expression (Blue: downregulated genes, red: upregulated genes, gray: genes with no significant difference in expression levels). C) Enrichment analysis of upregulated KEGG pathways related to osteogenic regulation. D) GO enrichment analysis. (For interpretation of the references to color in this figure legend, the reader is referred to the Web version of this article.)

Bioinformatics analysis of RNA sequencing was performed to elucidate the osteogenic mechanism triggered by photothermal stimulation within the GM/PRF-C scaffold. Analysis of differentially expressed genes (DEGs) identified 1034 upregulated and 359 downregulated genes in the GM/PRF-C + NIR group compared with control group (Fig. 9A and B). Gene Ontology enrichment analysis demonstrated that upregulated genes were significantly enriched in biological processes and molecular functions critical to bone formation, such as cell differentiation, cellular developmental processes, ossification, bone mineralization, extracellular matrix organization and cell adhesion (Fig. 9D). Key molecular functions included signaling receptor binding such as the BMP signaling pathway and cellular response to transforming growth factor beta stimulus. KEGG pathway analysis of the upregulated DEGs revealed significant enrichment of multiple osteogenesis-related pathways in the GM/PRF-C + NIR group (Fig. 9C). These included the PI3K-Akt signaling pathway, focal adhesion and ECM-receptor interaction, which were essential for cell survival, proliferation and integration with the extracellular environment. Additionally, Bone metabolism, such as the calcium signaling pathway and mineral absorption, were enriched in pathway analysis.

## 4 Discussion

The regeneration of infected bone defects represents a significant clinical challenge, primarily due to the high susceptibility of conventional implants to bacterial colonization [[35], [36], [37]]. In this study, we developed a 3D-printed GM hydrogel functionalized with PDA and PRF through either surface adsorption or coprecipitation, illustrating that the mode of incorporation strongly affected scaffold performance. Compared with surface adsorption, coprecipitation led to more homogeneous PDA/PRF integration within the hydrogel matrix, resulting in more favorable physicochemical properties, more sustained growth-factor release and superior osteogenic performance. These findings highlighted the importance of functionalization strategy in designing multifunctional scaffolds for infected bone regeneration.

The different integration modes directly influenced scaffold structure and function. Surface adsorption, involving sequential modification where pre-formed GM scaffold was first coated with PDA through self-polymerization and followed by PRF immobilization via molecular interactions, resulted in predominantly surface-localized distribution of bioactive components. Coprecipitation enabled simultaneous integration of PDA and PRF during hydrogel preparation, creating a homogeneous composite network where PRF became embedded within the hydrogel matrix rather than merely surface-bound. This homogeneous internal distribution improved component retention and helped maintain its function during degradation, which is particularly important for bone repair. These results were also consistent with our previous studies, in which surface adsorption and coprecipitation led different loading behaviors, release profiles and osteogenic outcomes in other biomaterial systems [28,29]. Thus, the novelty of the present work lied not only in constructing a PDA-based photothermal scaffold, but also in exhibiting the functionalization strategy itself was crucial for overall performance in the GM/PDA/PRF system.

Our study successfully established a dual-phase NIR irradiation strategy that enabled a precise transition from immediate antibacterial action to sustained osteogenic promotion through parameter modulation [[38], [39], [40]]. During the early antibacterial phase, high-power NIR irradiation rapidly increased the temperature of PDA-containing scaffolds bactericidal levels, effectively eradicating both *S. aureus* and *E. coli* [41,42]. During the later osteogenic phase, mild photothermal stimulation at 1 W/cm^2^ maintained temperatures around 40-42 °C, which was favorable for osteogenic differentiation. Generally, PDA primarily converts absorbed NIR energy into heat, and the local temperature of the PDA-containing hydrogels reached approximately 50-55 °C under irradiation [43]. Although PDA has been reported to possess ROS-scavenging activities, a range of 50-55 °C is sufficient to induce bacterial membrane or protein damage that kill bacteria effectively [44,45]. This explanation was also consistent with the significant reduction in bacterial viability and the disrupted bacterial morphology after NIR treatment.

In addition to antibacterial activity, NIR irradiation also triggered on-demand release of PRF-derived factors. Periodic NIR irradiation temporarily disrupted hydrogen bonding and hydrophobic interactions between PDA and PRF, triggering precise growth factors release at predetermined time points, which was evidenced by the significantly higher cumulative release of growth factors from NIR-irradiated GM/PDA/PRF compared to its non-irradiated counterpart [46]. GM/PRF-C showed more stable and prolonged release than GM/PRF-A, indicating that coprecipitation provided a more robust growth-factor delivery platform. It should also be noted that only PDGF-BB and TGF-β1 were quantified in the present study. Although they are representative PRF-derived factors related to bone regeneration, they do not fully reflect the complete biological profile of PRF. Broader profiling of PRF-derived bioactive molecules will therefore be included in future studies.

Compared with previously reported photothermal antibacterial platforms (i.e., gold nanoparticle-, black phosphorus-, or MXene-based materials), the present system offered several practical advantages [[47], [48], [49]]. It integrated PDA-mediated photothermal antibacterial activity, PRF-based regenerative support and a degradation profile matched to early bone repair within a single 3D-printed scaffold. More importantly, coprecipitation led to more stable PRF incorporation and better coupling of antibacterial and regenerative functions than surface adsorption, providing a more suitable design for infected bone defect repair.

Consistent with these design advantages, all groups demonstrated favorable cytocompatibility, indicating the basic biosafety of the GM-based hydrogel. In detail, the slight reduction in cell proliferation in the GM/PRF-A group might be attributed to the heterogeneous distribution of PDA, which could result in localized overheating and induce reversible thermal stress under irradiation. In contrast, the homogeneous integration of PDA/PRF achieved through coprecipitation in GM/PRF-C enabled to control temperature favored for cell activity under low-intensity NIR irradiation [50,51].

The superior osteogenesis effect of GM/PRF-C + NIR was evidenced by significantly elevated ALP activity, enhanced mineralized nodule formation, and upregulated expression of osteogenic markers. Transcriptomic analysis further revealed significant enrichment in biological processes related to multicellular organismal process, indicating the structure and function of GM/PRF-C could support complex tissue-level organization and cellular communication. Additionally, the significant enrichment of collagen-containing extracellular matrix component demonstrated the GM/PRF-C scaffold enhanced extracellular matrix deposition and maturation, which was a fundamental prerequisite for successful bone formation [52,53]. The enrichment in response to external stimulus further reflected the composite scaffold was sensitive to surrounding microenvironment, particularly in responding to photothermal stimulation and biochemical cues. More significantly, the activated cytokine-cytokine receptor interaction pathway directly corresponded to the controlled release of growth factors from the PRF components, suggesting that the GM/PRF-C scaffold could effectively orchestrate cytokine signaling networks essential for bone regeneration. The concurrent activation of extracellular matrix organization and cytokine-mediated signaling created a powerful synergistic microenvironment that promoted stem cell recruitment, proliferation and differentiation [[54], [55], [56]].

Finally, *in vivo* evaluation using a rat infected calvarial defect model confirmed the dual functionality of GM/PRF-C under inflammatory condition. Photothermal antibacterial therapy was applied during the first week post-implantation effectively cleared bacterial infection and controlled inflammation, establishing a sterile microenvironment that favored for regeneration. Subsequent mild NIR stimulation synergized with the sustained growth factors release to significantly enhance bone repair, as evidenced by micro-CT analysis at both 4 and 8 weeks. Histological examination further confirmed enhanced collagen deposition, matrix maturation and strong osteocalcin expression.

## 5 Conclusion

Our study presented a 3D-printed GM/PDA/PRF scaffold that combined NIR-triggered photothermal antibacterial activity with PRF-mediated regenerative capacity for infected bone repair. Compared with surface adsorption, the coprecipitation strategy enabled more stable PRF incorporation and led to better physicochemical properties, antibacterial efficacy and osteogenic performance. The resulting scaffold is therefore a promising strategy for infected bone defects, particularly those with irregular geometry that require both early infection control and subsequent regenerative support. Future studies will evaluate its performance in large-animal models, optimize the NIR treatment protocol and defect-specific scaffold design, and assess its long-term translational feasibility in clinically relevant settings.

## CRediT authorship contribution statement

**Zihang Yu:** Data curation, Methodology, Writing – original draft. **Lei Miao:** Data curation, Writing – original draft. **Shuchang Liu:** Data curation, Methodology. **Lijun Zhang:** Methodology. **Xiaoqing Wang:** Methodology. **Yujia Han:** Data curation. **Ziwen Cao:** Data curation. **Fan Liu:** Conceptualization, Funding acquisition, Supervision, Writing – review & editing. **Zhongti Zhang:** Funding acquisition, Supervision. **Yulou Tian:** Funding acquisition, Supervision.

## Declaration of competing interest

The authors declare that they have no known competing financial interests or personal relationships that could have appeared to influence the work reported in this paper.

## Data Availability

Data will be made available on request.
