# Supplementary material for: The coprecipitation-functionalized 3D-printed GM/PDA/PRF hydrogel for infected bone regeneration via synergistic photothermal antibacterial and osteogenic activity
Source: Mater Today Bio. 2026 May 14;38:103233. doi: 10.1016/j.mtbio.2026.103233 (PMC13199900; doi:10.1016/j.mtbio.2026.103233)
Supplement: Multimedia component 1 [file mmc1.docx]

Supplementary Information

**The coprecipitation-functionalized 3D-printed GM/PDA/PRF hydrogel for infected bone regeneration via synergistic photothermal antibacterial and osteogenic activity**

**Supplementary Methods**

1. Preparation of GM/PRF and GM/PDA/PRF scaffolds

To fabricate GM/PRF scaffolds, PRF powder was blended into the GM precursor solution (5% GelMA and 8% MC) at concentrations of 0.5%, 1.0% and 1.5% (w/v), corresponding to the groups denoted as GM/PRF₁, GM/PRF₂ and GM/PRF₃, respectively. Each mixture was homogenized by magnetic stirring at 37 °C for 3 h.

For GM/PDA/PRF scaffolds, pre-synthesized PDA/PRF powder was incorporated into the GM precursor at a fixed PRF concentration of 1% (w/v) with different PDA contents of 0.05%, 0.1% or 0.15% (w/v), known as GM/PDA₁/PRF₂, GM/PDA₂/PRF₂ and GM/PDA₃/PRF₂ groups. Each composite precursor was similarly homogenized by magnetic stirring at 37 °C for 3 h.

All precursors were subsequently printed into scaffolds using a desktop bioprinter (SunP BioMarker 2i, China) with a 400 μm nozzle at 4 °C, an extrusion pressure of 120 kPa, and a printing speed of 2 mm/s (line spacing: 1 mm; layer height: 0.3 mm). The printed constructs were immediately photo-crosslinked under UV light (25 mW/cm²) for 60 s.

2. The optimal PDA/PRF concentration

BMSCs were seeded onto sterilized scaffolds, and cell proliferation was assessed via CCK‑8 kit for three days. Alkaline phosphatase (ALP) activity was measured spectrophotometrically on days 3 and 7, with values normalized to the total protein content. After identifying the optimal PRF concentration, cell viabilities and ALP activities of PDA‑varied groups were investigated based on the protocol above.

3. Optimal irradiation density

To optimize irradiation parameters, additional multi-power photothermal tests were performed at 0.5, 1.0 and 2.0 W/cm². The temperatures were recorded with an infrared thermal camera (Hikmicro, China) for 5 min. The optimal irradiation density was assessed based on the evaluated temperature and the following post-irradiation cytocompatibility. In brief, cell viability was assessed using the CCK‑8 kit for 48 h.

**Supplementary Figures**


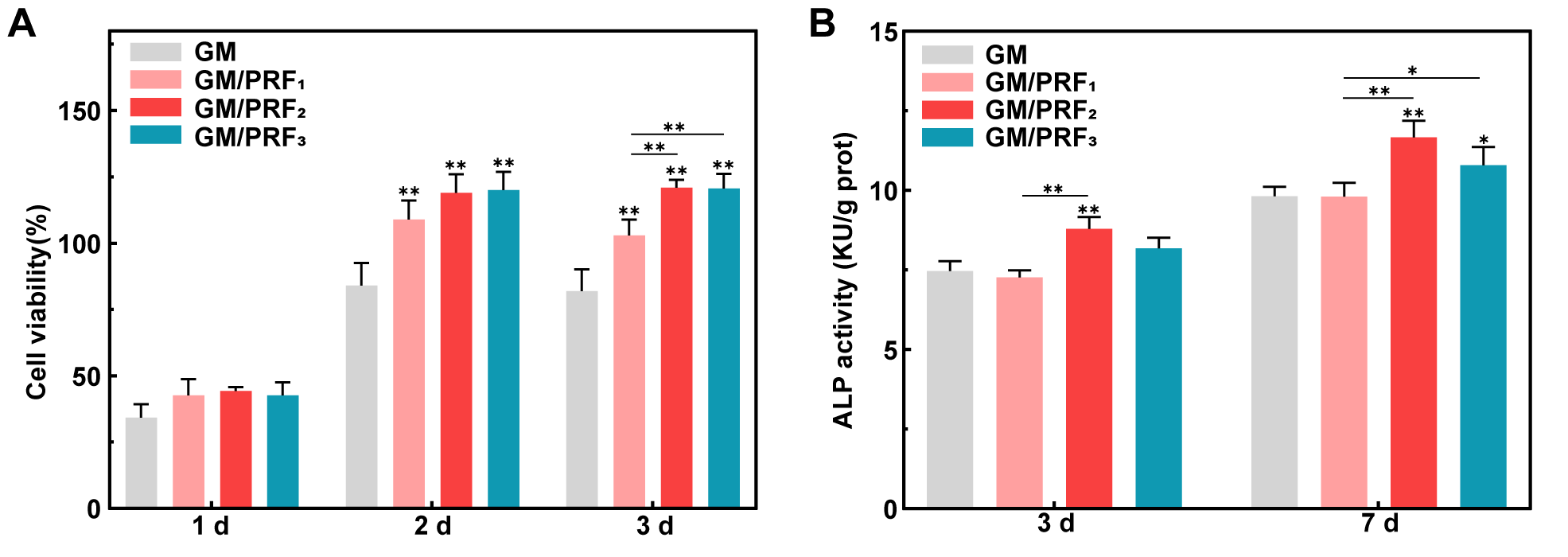


**Figure S1. *In vitro* biological performance of GM and GM/PRF composite hydrogels.** A) Cell viabilities. B) ALP activities. (**p* < 0.05, ***p* < 0.01, N = 3).

**
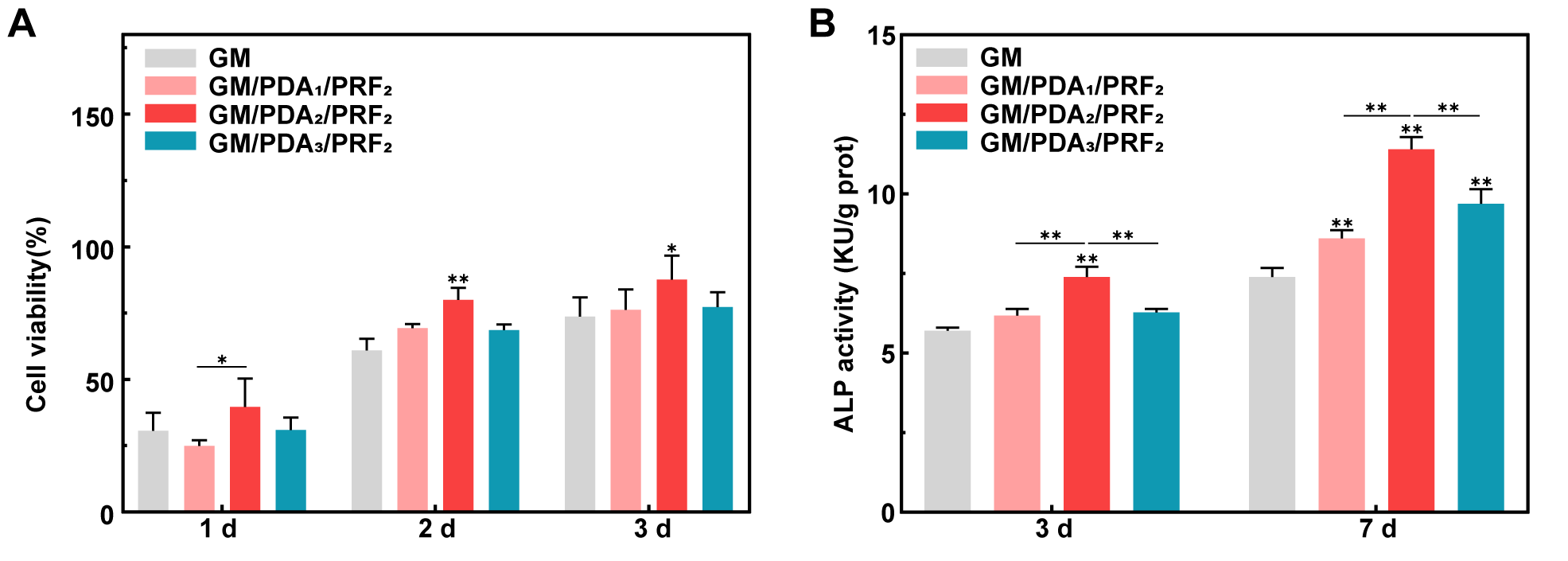
**

**Figure S2. *In vitro* biological performance of GM and GM/PDA/PRF composite hydrogels.** A) Cell viabilities. B) ALP activities. (**p* < 0.05, ***p* < 0.01, N = 3).


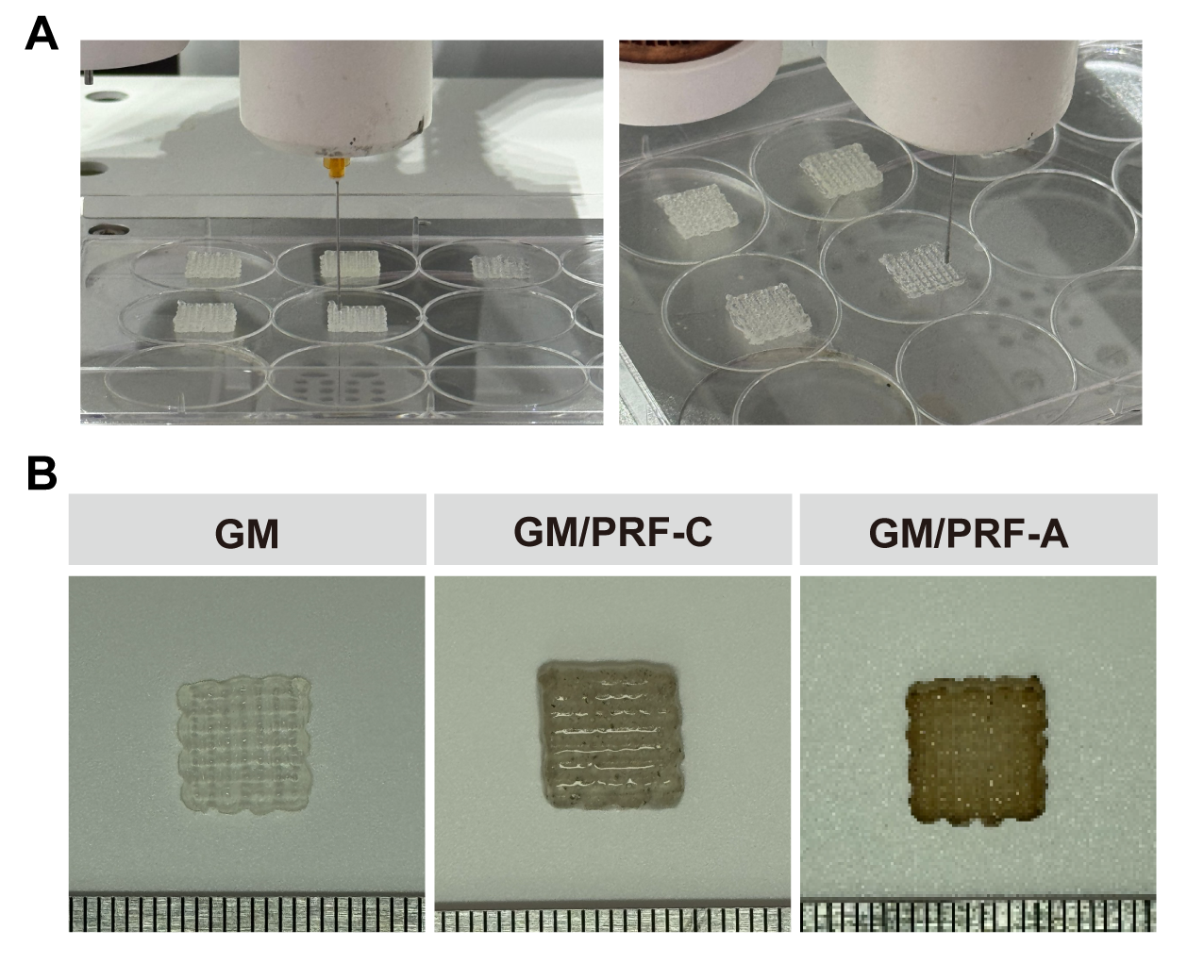


**Figure S3. General observation of the printed scaffolds**. A) Printing process. B) Representative images of the printed scaffolds.


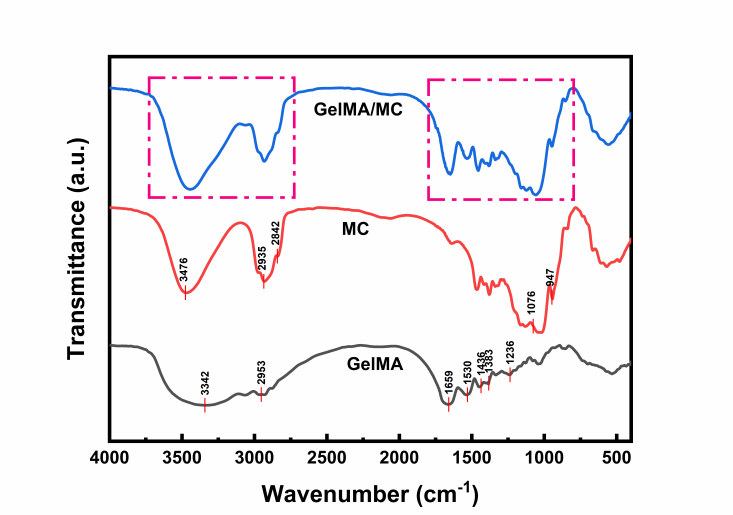


**Figure S4. FTIR spectra of GelMA, MC and GelMA/MC.**

**Figure S5. EDS analysis of the composite hydrogels** A) GM. B) GM/PRF-C. C) GM/PRF-A. (Scale bar = 100 μm).


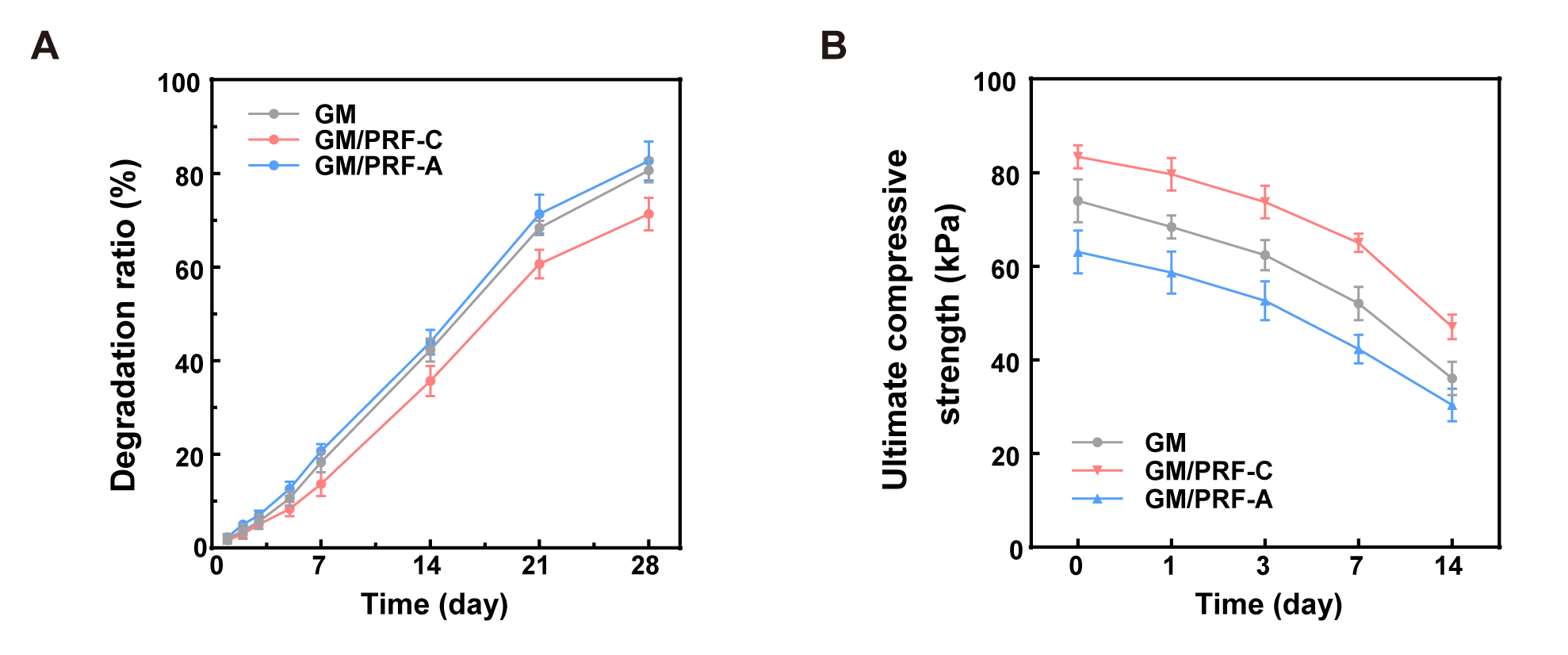


**Figure S6. Physical properties of the composite hydrogels under infection-relevant conditions.** A) Degradation behavior. B) Compressive property. (**p* < 0.05, ***p* < 0.01, N = 3).


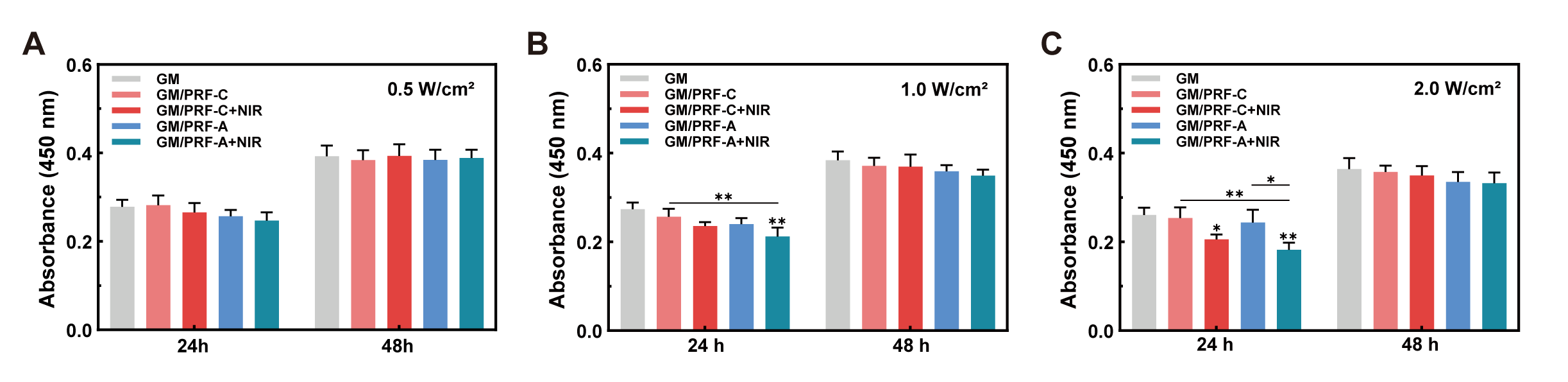


**Figure S7. *In vitro* cell viability under NIR irradiation at different power densities.** A) 0.5 W/cm², B) 1.0 W/cm², and C) 2.0 W/cm². Cell viability was assessed at day 1 and day 2 post-irradiation. (**p* < 0.05, ***p* < 0.01, N = 3).


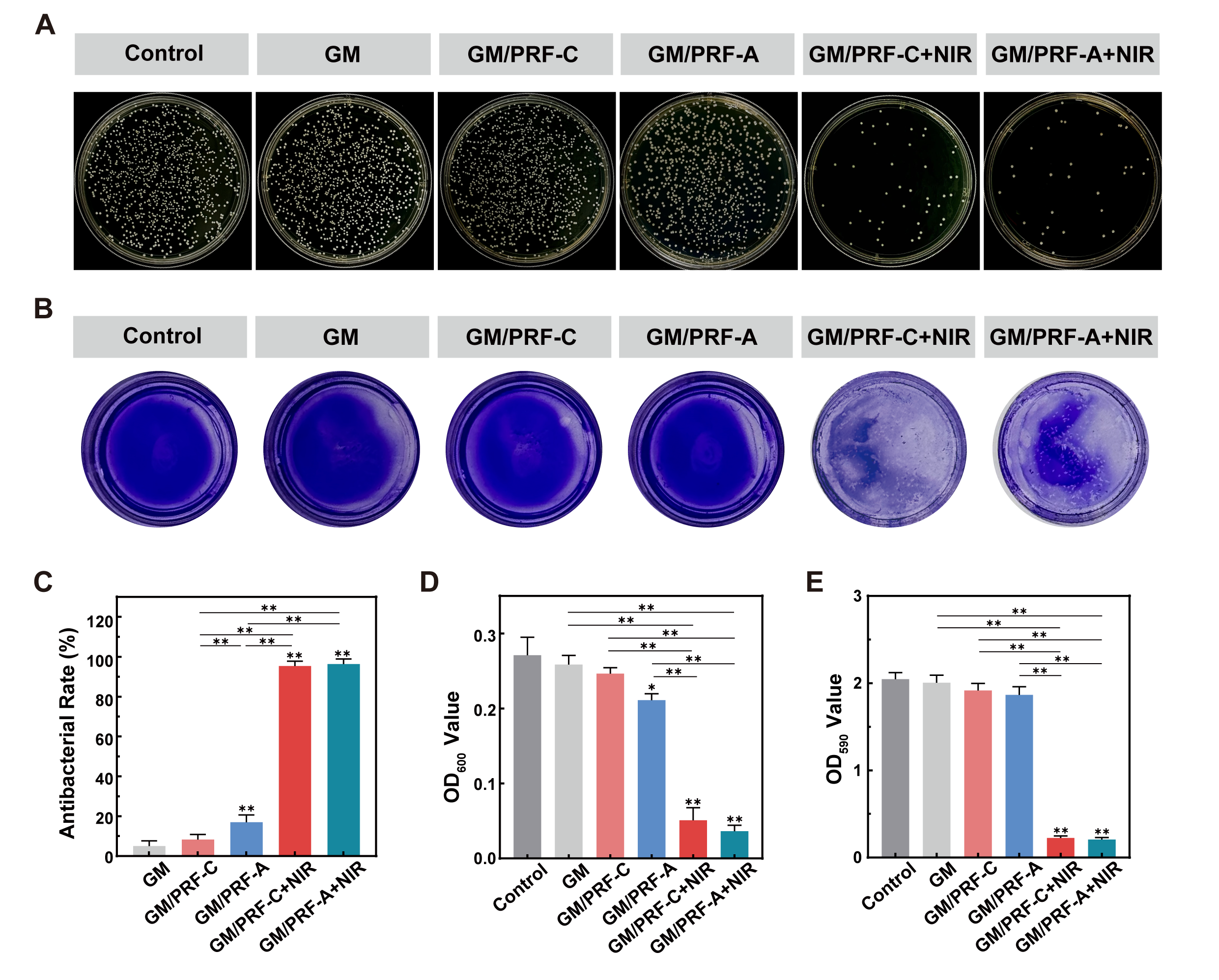


**Figure S8. *In vitro* antibacterial evaluation of the composite hydrogels against MRSA.** A) Representative images of bacterial colonies in the presence of composite hydrogels with or without NIR irradiation (2 W/cm², 5 min). B) Crystal violet staining of biofilm. C) Quantitative antibacterial rates of composite hydrogels with or without NIR irradiation against MRSA. D) Bacterial growth kinetics of MRSA. E) Quantitative analysis of crystal violet‑stained biofilms. (**p* < 0.05, ***p* < 0.01, N = 3).


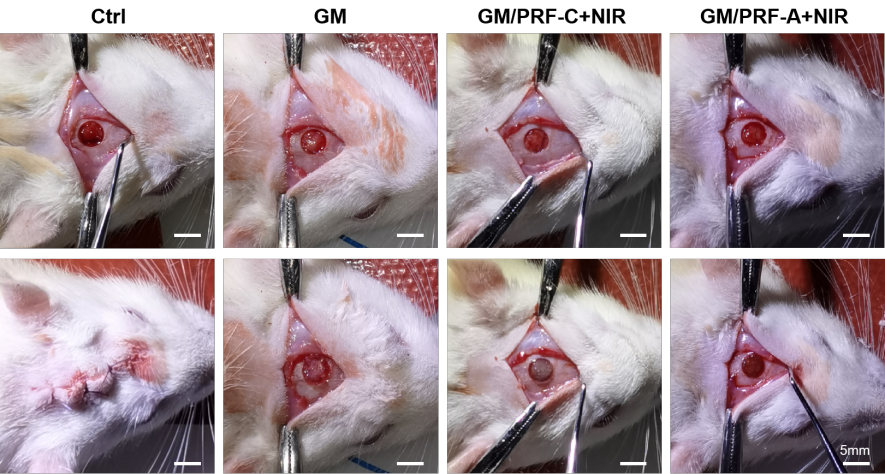


**Figure S9. Schematic illustration of the infected bone defect model establishment and the composite hydrogels implantation.** (Scale bar = 5 mm).


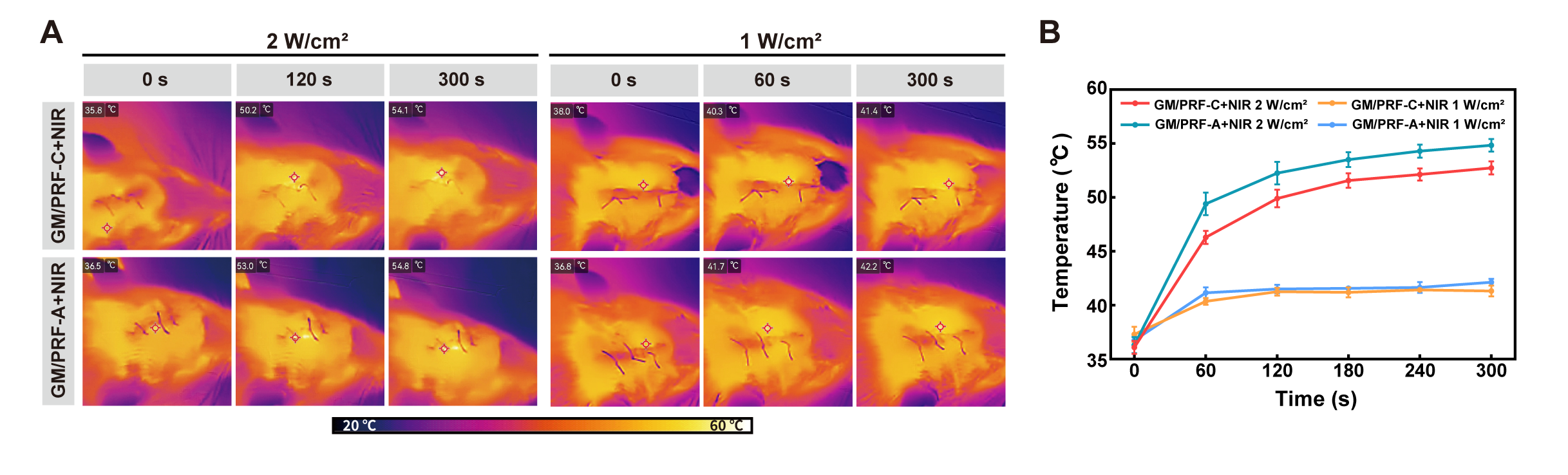


**Figure S10. Photothermal performance of the composite hydrogels *in vivo*.** A) Representative infrared thermographic images. B) Temperature variation of GM/PRF-C+NIR and GM/PRF-A+NIR groups (808 nm, 2.0 W/cm² or 1.0 W/cm²) *in vivo.* (**p* < 0.05, ***p* < 0.01, N = 6).


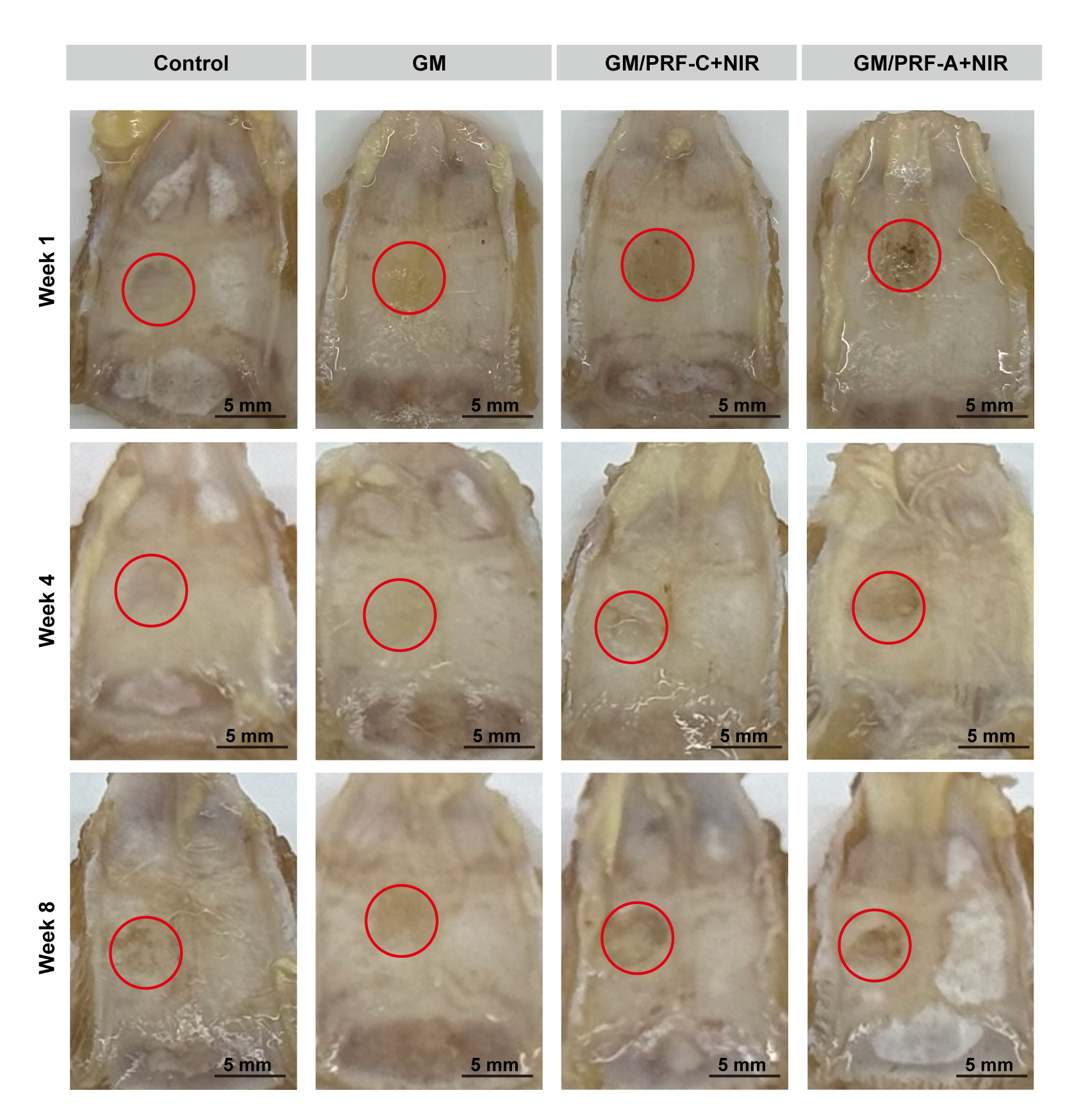


**Figure S11**. **Representative images of cranial defects at 1-, 4- and 8-weeks post-implantation.** (Scale bar = 5 mm).


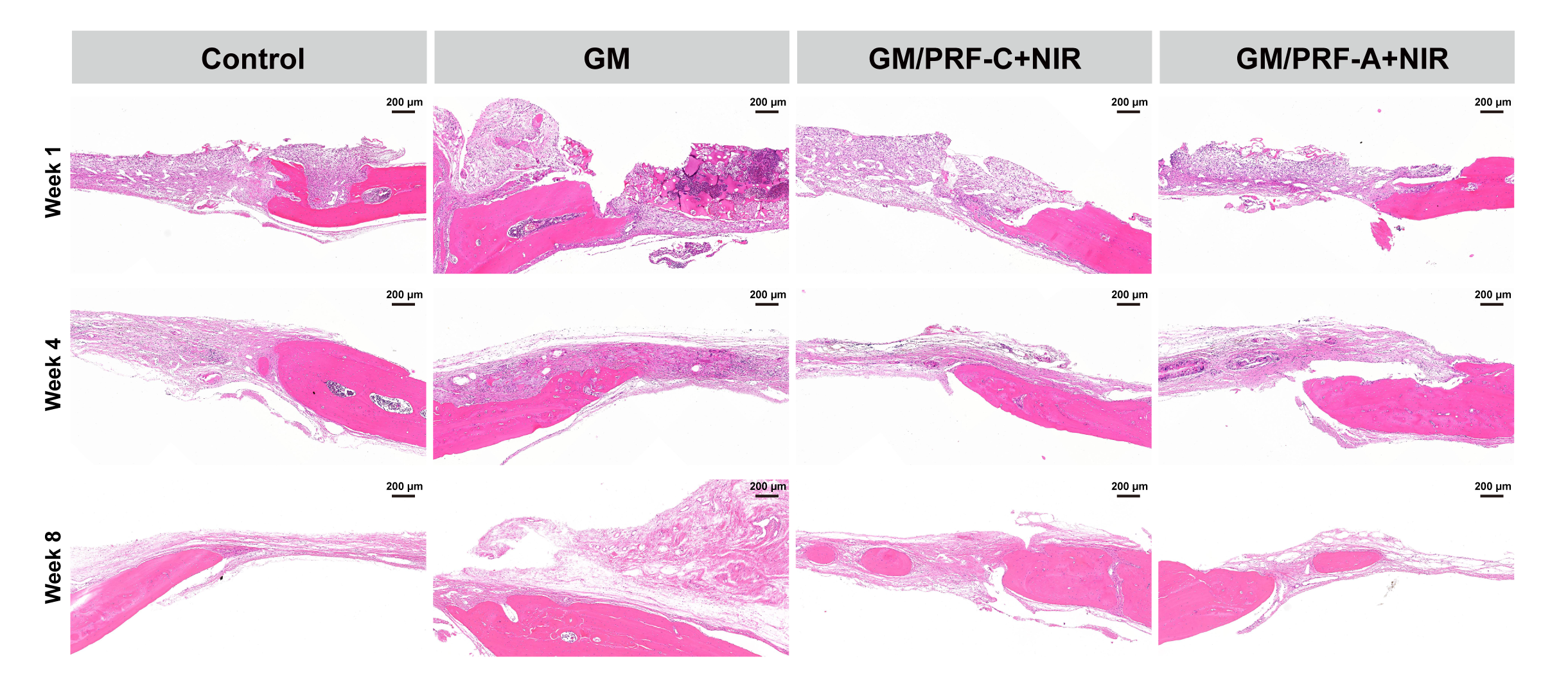


**Figure S12. Representative images of H&E staining in the local soft tissues surrounding the infected calvarial defect at 1-, 4- and 8-weeks post-implantation.** (Scale bar = 200 μm).


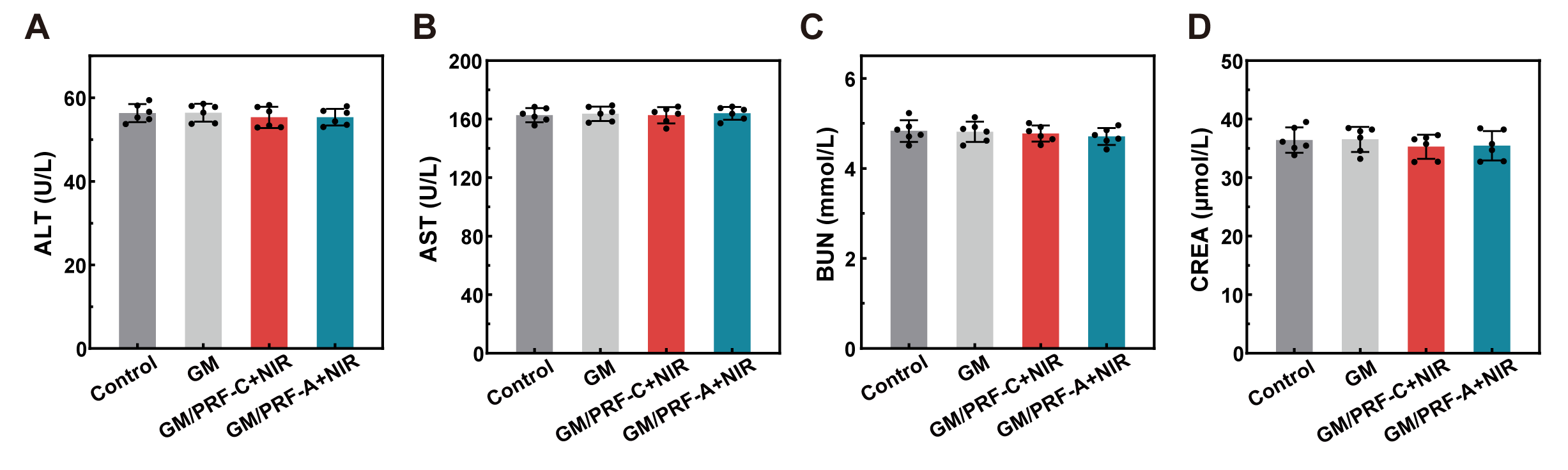


**Figure. S13. *In vivo* biosafety evaluation of the composite hydrogels.** A) Alanine aminotransferase (ALT). B) Aspartate aminotransferase (AST). C) Blood urea nitrogen (BUN). D) Creatinine (Cr). (**p* < 0.05, ***p* < 0.01, N = 6).


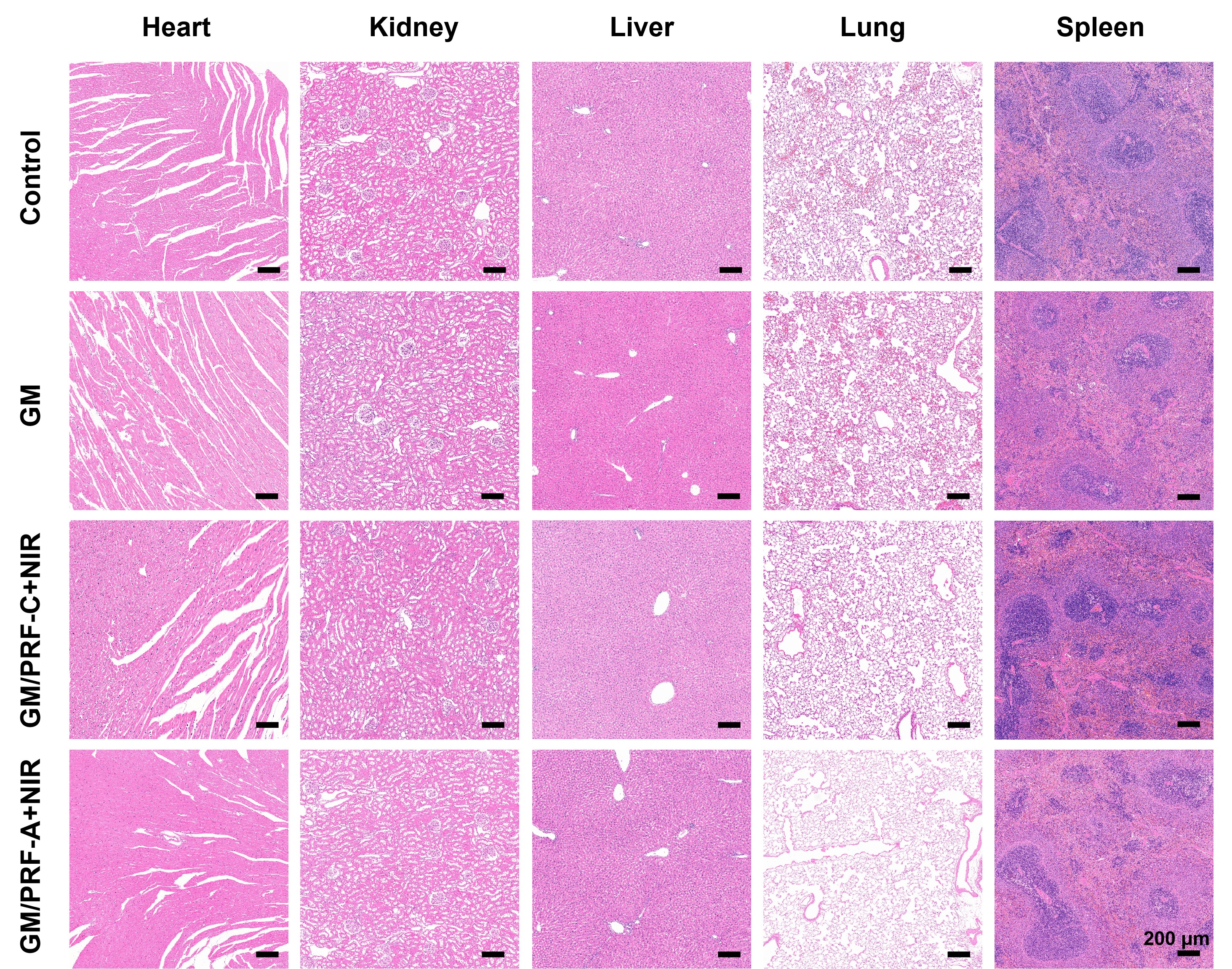


**Figure S14. Representative images of H&E staining for major organs after 8 weeks of implantation.** (Scale bar = 200 μm).

**Supplementary Table**

**Table S1.** Primer used for qPCR of osteogenic factors.

| **Gene** | **Forward primers** | **Reverse primers** |
| --- | --- | --- |
| *Col Ⅰ* | TGTTGGTCCTGCTGGCAAGAATG | GTCACCTTGTTCGCCTGTCTCAC |
| *OCN* | GGACCCTCTCTCTGCTCACTCTG | ACCTTACTGCCCTCCTGCTTGG |
| *OPN* | TGGAAACACACAGCCTGGAG | CGCCTGACTGTCGATAGCAT |
| *OSX* | GCCTACTTACCCGTCTGACTTTGC | CCCTCCAGTTGCCCACTATTGC |
| *Gapdh* | ACGGCAAGTTCAACGGCACAG | CGACATACTCAGCACCAGCATCAC |
